# Cell Specific eQTL Analysis without Sorting Cells

**DOI:** 10.1371/journal.pgen.1005223

**Published:** 2015-05-08

**Authors:** Harm-Jan Westra, Danny Arends, Tõnu Esko, Marjolein J. Peters, Claudia Schurmann, Katharina Schramm, Johannes Kettunen, Hanieh Yaghootkar, Benjamin P. Fairfax, Anand Kumar Andiappan, Yang Li, Jingyuan Fu, Juha Karjalainen, Mathieu Platteel, Marijn Visschedijk, Rinse K. Weersma, Silva Kasela, Lili Milani, Liina Tserel, Pärt Peterson, Eva Reinmaa, Albert Hofman, André G. Uitterlinden, Fernando Rivadeneira, Georg Homuth, Astrid Petersmann, Roberto Lorbeer, Holger Prokisch, Thomas Meitinger, Christian Herder, Michael Roden, Harald Grallert, Samuli Ripatti, Markus Perola, Andrew R. Wood, David Melzer, Luigi Ferrucci, Andrew B. Singleton, Dena G. Hernandez, Julian C. Knight, Rossella Melchiotti, Bernett Lee, Michael Poidinger, Francesca Zolezzi, Anis Larbi, De Yun Wang, Leonard H. van den Berg, Jan H. Veldink, Olaf Rotzschke, Seiko Makino, Veikko Salomaa, Konstantin Strauch, Uwe Völker, Joyce B. J. van Meurs, Andres Metspalu, Cisca Wijmenga, Ritsert C. Jansen, Lude Franke

**Affiliations:** 1 University of Groningen, University Medical Center Groningen, Department of Genetics, Groningen, The Netherlands; 2 Groningen Bioinformatics Centre, University of Groningen, Groningen, The Netherlands; 3 Estonian Genome Center, University of Tartu, Tartu, Estonia; 4 Divisions of Endocrinology, Boston Children's Hospital, Boston, Massachusetts, United States of America; 5 Department of Genetics, Harvard Medical School, Boston, Massachusetts, United States of America; 6 Broad Institute, Cambridge, Massachusetts, United States of America; 7 Department of Internal Medicine, Erasmus Medical Centre Rotterdam, the Netherlands; 8 The Netherlands Genomics Initiative-sponsored Netherlands Consortium for Healthy Aging (NGI-NCHA), Leiden/ Rotterdam, the Netherlands; 9 Interfaculty Institute of Genetics and Functional Genomics, University Medicine Greifswald, Greifswald, Germany; 10 The Charles Bronfman Institute for Personalized Medicine, Genetics of Obesity & Related Metabolic Traits Program, Icahn School of Medicine at Mount Sinai, New York, New York, United States of America; 11 Institute of Human Genetics, Helmholtz Zentrum München, German Research Center for Environmental Health, Neuherberg, Germany; 12 Institut für Humangenetik, Technische Universität München, München, Germany; 13 Computational Medicine, Institute of Health Sciences, Faculty of Medicine, University of Oulu, Oulu, Finland; 14 Institute for Molecular Medicine Finland FIMM, University of Helsinki, Helsinki, Finland; 15 Department of Chronic Disease Prevention, National Institute for Health and Welfare, Helsinki, Finland; 16 Genetics of Complex Traits, University of Exeter Medical School, University of Exeter, Exeter, United Kingdom; 17 Wellcome Trust Centre for Human Genetics, Oxford, United Kingdom; 18 Department of Oncology, Cancer and Haematology Centre, Churchill Hospital, Oxford, United Kingdom; 19 Singapore Immunology Network (SIgN), Agency for Science, Technology and Research (A*STAR), Singapore; 20 University of Groningen, University Medical Center Groningen, Department of Gastroenterology and Hepatology, Groningen, The Netherlands; 21 Institute of Molecular and Cell Biology, University of Tartu, Tartu, Estonia; 22 Molecular Pathology, Institute of Biomedicine and Translational Medicine, University of Tartu, Tartu, Estonia; 23 Department of Epidemiology, Erasmus Medical Center Rotterdam, Rotterdam, the Netherlands; 24 Institute for Clinical Chemistry and Laboratory Medicine, University Medicine Greifswald, Greifswald, Germany; 25 Institute for Community Medicine, University Medicine Greifswald, Greifswald, Germany; 26 Institute of Human Genetics, Helmholtz Zentrum München, German Research Center for Environmental Health, Neuherberg, Germany; 27 Institut für Humangenetik, Technische Universität München, München, Germany; 28 Munich Heart Alliance, Munich, Germany; 29 German Center for Cardiovascular Research (DZHK), Germany; 30 Institute for Clinical Diabetology, German Diabetes Center, Leibniz Center for Diabetes Research at Heinrich Heine University Düsseldorf, Düsseldorf, Germany; 31 German Center for Diabetes Research (DZD), partner site Düsseldorf, Düsseldorf, Germany; 32 Department of Diabetology and Endocrinology, University Hospital Düsseldorf, Heinrich Heine University, Düsseldorf, Germany; 33 Research Unit of Molecular Epidemiology, Helmholtz Zentrum München, German Research Center for Environmental Health, Neuherberg, Germany; 34 Institute for Molecular Medicine Finland FIMM, University of Helsinki, Helsinki, Finland; 35 Department of Chronic Disease Prevention, National Institute for Health and Welfare, Helsinki, Finland; 36 Wellcome Trust Sanger Institute, Hinxton, Cambridge, United Kingdom; 37 Department of Public Health, Hjelt Institute, University of Helsinki, Helsinki, Finland; 38 Institute of Biomedical and Clinical Sciences, University of Exeter Medical School, Exeter, United Kingdom; 39 Clinical Research Branch, National Institute on Aging NIA-ASTRA Unit, Harbor Hospital, Baltimore, Maryland, United States of America; 40 Laboratory of Neurogenetics, National Institute on Aging, National Institutes of Health, Bethesda, Maryland, United States of America; 41 Department of Molecular Neuroscience and Reta Lila Laboratories, Institute of Neurology, UCL, London, United Kingdom; 42 Doctoral School in Translational and Molecular Medicine (DIMET), University of Milano-Bicocca, Milan, Italy; 43 Department of Otolaryngology, National University of Singapore, Singapore; 44 Department of Neurology, Rudolf Magnus Institute of Neuroscience, University Medical Centre Utrecht, Utrecht, The Netherlands; 45 Institute of Genetic Epidemiology, Helmholtz Zentrum München, German Research Center for Environmental Health, Neuherberg, Germany; 46 Institute of Medical Informatics, Biometry and Epidemiology, Chair of Genetic Epidemiology, Ludwig-Maximilians-Universität, Neuherberg, Germany; McGill University, CANADA

## Abstract

The functional consequences of trait associated SNPs are often investigated using expression quantitative trait locus (eQTL) mapping. While trait-associated variants may operate in a cell-type specific manner, eQTL datasets for such cell-types may not always be available. We performed a genome-environment interaction (GxE) meta-analysis on data from 5,683 samples to infer the cell type specificity of whole blood *cis*-eQTLs. We demonstrate that this method is able to predict neutrophil and lymphocyte specific *cis*-eQTLs and replicate these predictions in independent cell-type specific datasets. Finally, we show that SNPs associated with Crohn’s disease preferentially affect gene expression within neutrophils, including the archetypal NOD2 locus.

## Author Summary

Many variants in the genome, including variants associated with disease, affect the expression of genes. These so-called expression quantitative trait loci (eQTL) can be used to gain insight in the downstream consequences of disease. While it has been shown that many disease-associated variants alter gene expression in a cell-type dependent manner, eQTL datasets for specific cell types may not always be available and their sample size is often limited. We present a method that is able to detect cell type specific effects within eQTL datasets that have been generated from whole tissues (which may be composed of many cell types), in our case whole blood. By combining numerous whole blood datasets through meta-analysis, we show that we are able to detect eQTL effects that are specific for neutrophils and lymphocytes (two blood cell types). Additionally, we show that the variants associated with some diseases may preferentially alter the gene expression in one of these cell types. We conclude that our method is an alternative method to detect cell type specific eQTL effects, that may complement generating cell type specific eQTL datasets and that may be applied on other cell types and tissues as well.

## Introduction

In the past seven years, genome-wide association studies (GWAS) have identified thousands of genetic variants that are associated with human disease [1]. The realization that many of the disease-predisposing variants are non-coding and that single nucleotide polymorphisms (SNPs) often affect the expression of nearby genes (i.e. *cis*-expression quantitative trait loci; *cis*-eQTLs) [2] suggests these variants have a predominantly regulatory function. Recent studies have shown that disease-predisposing variants in humans often exert their regulatory effect on gene expression in a cell-type dependent manner [3–5]. However, most human eQTL studies have used sample data obtained from mixtures of cell types (e.g. whole blood) or a few specific cell types (e.g. lymphoblastoid cell lines) due to the prohibitive costs and labor required to purify subsets of cells from large samples (by cell sorting or laser capture micro-dissection). In addition, the method of cell isolation can trigger uncontrolled processes in the cell, which can cause biases. In consequence, it has been difficult to identify in which cell types most disease-associated variants exert their effect.

**Fig 1:**
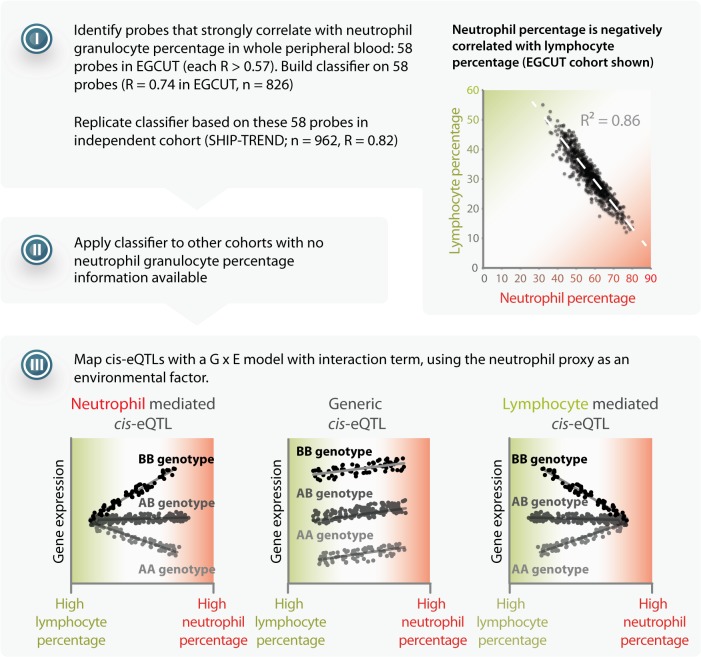
Method overview. I) Starting with a dataset that has cell count measurements, determine a set of probes that have a strong positive correlation to the cell count measurements. Calculate the correlation between these specific probes in the other datasets, and apply principal component analysis to combine them into a single proxy for the cell count measurement. II) Apply the prediction to other datasets lacking cell count measurements. III) Use the proxy as a covariate in a linear model with an interaction term in order to distinguish cell-type-mediated from non-cell-type-mediated eQTL effects.

Here we describe a generic approach that uses eQTL data in mixtures of cell types to infer cell-type specific eQTLs (Fig 1). Our strategy includes: (i) collecting gene expression data from an entire tissue; (ii) predicting the abundance of its constituent cell types (i.e. the cell counts), by using expression levels of genes that serve as proxies for these different cell types (since not all datasets might have actual constituent cell count measurements). We used an approach similar to existing expression and methylation deconvolution methods [6–11]; (iii) run an association analysis with a term for interaction between the SNP and the proxy for cell count to detect cell-type-mediated or-specific associations, and (iv) test whether known disease associations are enriched for SNPs that show the cell-type-mediated or-specific effects on gene expression (i.e. eQTLs).

## Results

We applied this strategy to 5,863 unrelated, whole blood samples from seven cohorts: EGCUT[12], InCHIANTI [13], Rotterdam Study [14], Fehrmann [2], SHIP-TREND [15], KORA F4 [16], and DILGOM [17]. Blood contains many different cell types that originate from either the myeloid (e.g. neutrophils and monocytes) or lymphoid lineage (e.g. B-cells and T-cells). Even though neutrophils comprise ~60% [18,19] of all white blood cells, no neutrophil eQTL data have been published to date, because this cell type is particularly difficult to purify or culture in the lab [20].

**Fig 2:**
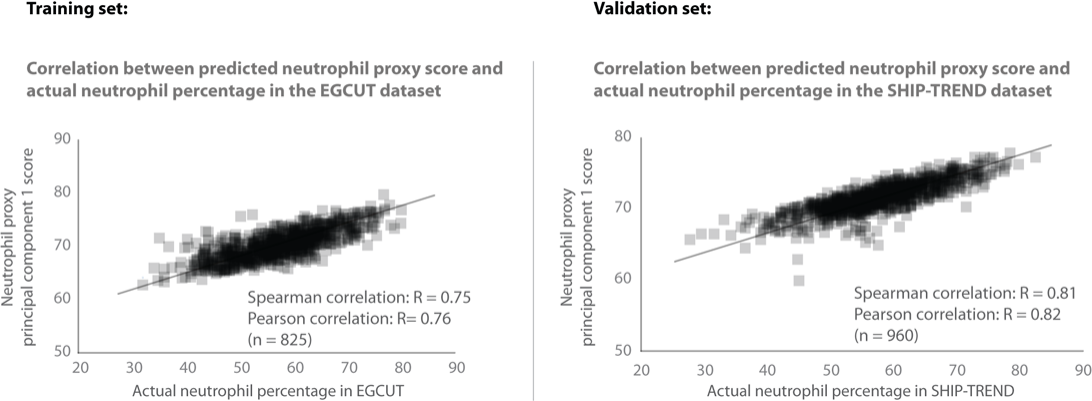
Validation of neutrophil proxy. There is a strong correlation between the neutrophil proxy and the actual neutrophil percentage measurements in the training dataset (EGCUT, Spearman R = 0.75). Validation of neutrophil prediction in the SHIP-TREND cohort shows a strong correlation (Spearman R = 0.81) between the neutrophil proxy and actual neutrophil percentage measurements in this dataset.

For the purpose of illustrating our cell-type specific analysis strategy in the seven whole blood cohorts, we focused on neutrophils. Direct neutrophil cell counts and percentages were only available in the EGCUT and SHIP-TREND cohorts, requiring us to infer neutrophil percentages for the other five cohorts. We used the EGCUT cohort as a training dataset to identify a list of 58 Illumina HT12v3 probes that correlated positively with neutrophil percentage (Spearman’s correlation coefficient R > 0.57; S1 Fig, S1 Table). We observed that 95% of these genes show much higher expression in purified neutrophils, as compared to 13 other purified blood cell-types, based on RNA-seq data from the BLUEPRINT epigenome project [21] (S2 Fig). We then summarized the gene expression levels of these 58 individual probes into a single neutrophil percentage estimate, by applying principal component analysis (PCA) and determining the first principal component (PC). We then used this first PC as a proxy for neutrophil percentage, an approach that is similar to existing expression and methylation deconvolution methods [6–11]. In the EGCUT dataset, the actual neutrophil percentage showed some correlation with age (Pearson R = 0.08, P = 0.02), but no association with gender (Student's T-test P = 0.31; S3 Fig) in the EGCUT dataset. The proxy for neutrophil percentage showed the same behavior: some correlation with age (Pearson R = 0.14, P = 6 x 10^–5^) and no association with gender (P = 0.11). This predicted neutrophil percentage strongly correlated with the actual neutrophil percentage (Spearman R = 0.75, Pearson R = 0.76; Fig 2). Including more or fewer top probes than the top 58 probes resulted in similar correlations (S4 Fig). We then used this set of 58 probes in each of the other cohorts as well, and used PCA per cohort on the probe correlation matrix of these 58 probes. In the SHIP-TREND cohort, for which the actual neutrophil percentage was available as well, we observed that the inferred neutrophil proxy strongly correlated with the actual neutrophil percentage (Spearman R = 0.81, Pearson R = 0.82; Fig 2).

Here we limited our analysis to 13,124 *cis*-eQTLs that were previously discovered in a whole blood eQTL meta-analysis of a comparable sample size [22] (we note that these 13,124 *cis*-eQTLs were detected while assuming a generic effect across cell-types, and as such, genome-wide application of cell-type specificity strategy might result in the detection of additional cell-type-specific *cis*-eQTLs). To infer the cell-type specificity of each of these eQTLs, we performed the eQTL association analysis with a term for interaction between the SNP marker and the proxy for cell count within each cohort, followed by a meta-analysis of the interaction term (weighted for sample size) across all the cohorts. We identified 1,117 *cis*-eQTLs with a significant interaction effect (8.5% of all *cis-*eQTLs tested; false discovery rate (FDR) < 0.05; 1,037 unique SNPs and 836 unique probes; S2 Table and S3 Table). The power to detect these effects depends on the original *cis*-eQTL effect size, as we observed that the main effect of *cis*-eQTLs that have a significant interaction term generally explained more variance in gene expression in the previously published *cis*-eQTL meta-analysis [22], as compared to the *cis*-eQTLs that did not show a significant interaction: 71% of the cell-type specific eQTLs had a main effect that explained at least 3% of the total expression variation, whereas 25% of the *cis*-eQTLs that did not show a show significant interaction explained at least 3% of the total expression variation (S5 Fig). Out of the total number of *cis*-eQTLs tested, 909 (6.9%) had a positive direction of effect, which indicates that these *cis*-eQTLs show stronger effect sizes when more neutrophils are present (i.e. ‘neutrophil-mediated *cis*-eQTLs’; 843 unique SNPs and 692 unique probes). Another 208 (1.6%) had a negative direction of effect (196 unique SNPs and 145 unique probes), indicating a stronger *cis*-eQTL effect size when more lymphoid cells are present (i.e. ‘lymphocyte-mediated *cis*-eQTLs’; since lymphocyte percentages are strongly negatively correlated with neutrophil percentages; Fig 1). Overall, the directions of the significant interaction effects were consistent across the different cohorts, indicating that our findings are robust (S6 Fig).

**Fig 3:**
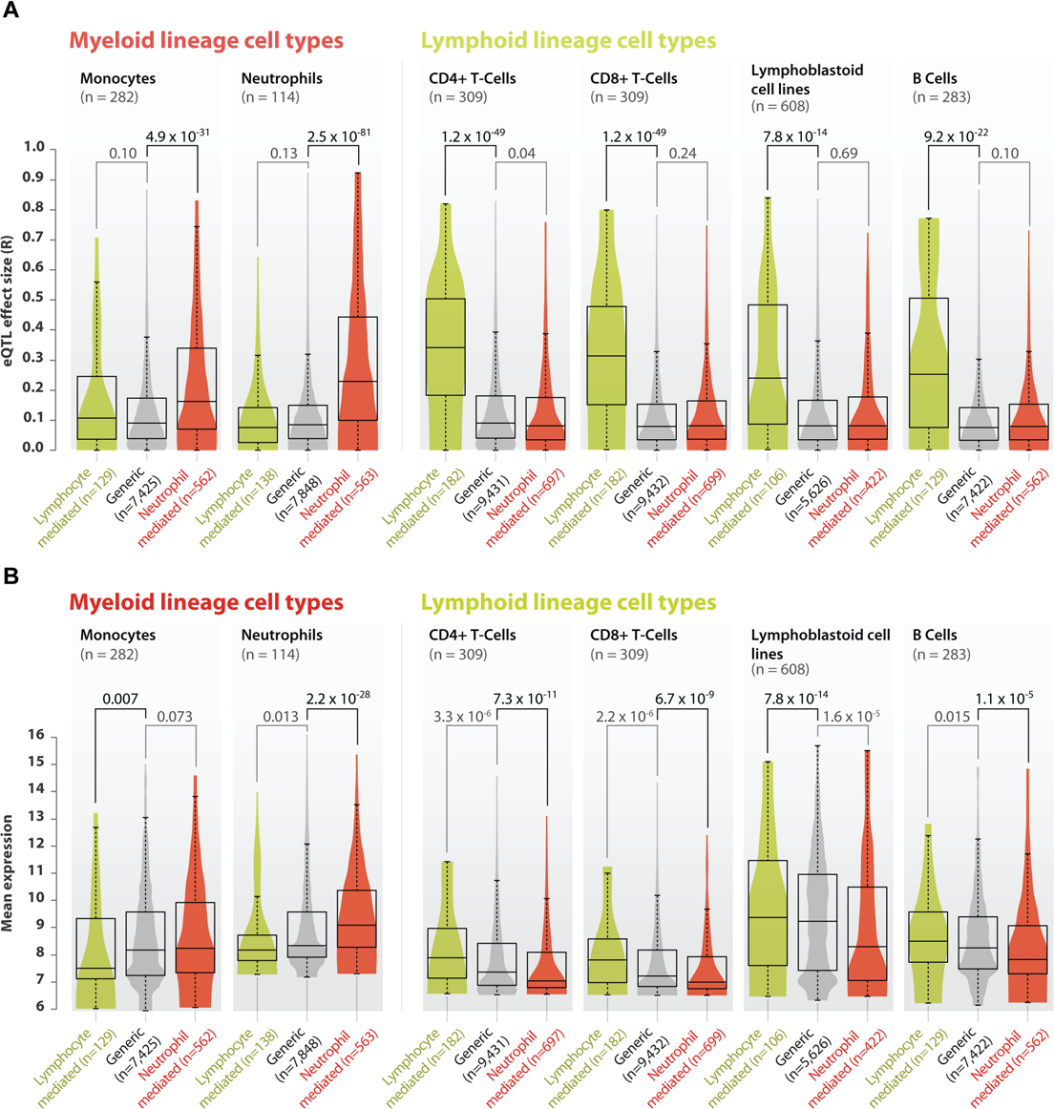
Validation of neutrophil and lymphoid specific *cis*-eQTLs in purified cell type eQTL datasets. A) We validated the neutrophil- and lymphoid-mediated *cis*-eQTL effects in four purified cell type datasets from the lymphoid lineage (B-cells, CD4+ T-cells, CD8+ T-cells and lymphoblastoid cell lines) and in two datasets from the myeloid lineage (monocytes and neutrophils). Compared to generic *cis*-eQTLs, large effect sizes were observed for neutrophil-mediated *cis*-eQTLs in myeloid lineage cell types, and small effect sizes in the lymphoid datasets. Conversely, lymphoid-mediated *cis*-eQTL effects had large effect sizes specifically in the lymphoid lineage datasets, while having smaller effect sizes in myeloid lineage datasets. These results indicate that our method is able to reliably predict whether a specific *cis*-eQTL is mediated by cell type. B) Comparison between average gene expression levels between different purified cell type eQTL datasets shows that neutrophil mediated *cis*-eQTLs have, on average a lower expression in cell types derived from the lymphoid lineage, and a high expression in myeloid cell types, while the opposite is true for lymphocyte mediated *cis*-eQTLs.

We validated the neutrophil- and lymphoid-mediated *cis*-eQTLs in six small, but purified cell-type gene expression datasets that had not been used in our meta-analysis. We generated new eQTL data from two lymphoid cell types (CD4+ and CD8+ T-cells) and one myeloid cell type (neutrophils, see online methods) and used previously generated eQTL data on two lymphoid cell types (lymphoblastoid cell lines and B-cells) and another myeloid cell type (monocytes, S4 Table). As expected, compared to *cis*-eQTLs without a significant interaction term (‘generic *cis*-eQTLs’, n = 12,007) the 909 neutrophil-mediated *cis*-eQTLs did indeed show very strong *cis*-eQTL effects in the neutrophil dataset (Wilcoxon P = 2.5 x 10^–81^ when compared to generic *cis-*eQTLs; Fig 3A). We observed that these *cis*-eQTLs also showed an increased effect-size in the monocyte dataset (Wilcoxon P-value = 4.9 x 10^–31^ when compared to generic *cis*-eQTLs; Fig 3A). Because both neutrophils and monocytes are myeloid lineage cells, this suggests that some of the neutrophil-mediated *cis*-eQTL effects also show stronger effects in other cells of the myeloid lineage. Conversely, the 208 lymphoid-mediated *cis*-eQTLs had a pronounced effect in each of the lymphoid datasets (Wilcoxon P-value ≤ 7.8 x 10^–14^ compared to generic *cis*-eQTLs; Fig 3A), while having small effect sizes in the myeloid datasets. These validation results indicate that our method is able to reliably predict whether a *cis*-eQTL is mediated by a specific cell type. Unfortunately, the cell type that mediates the *cis*-eQTL is not necessarily the one in which the *cis*-gene has the highest expression (Fig 3B), making it impossible to identify cell-type-specific eQTLs directly on the basis of expression levels.

Myeloid and lymphoid blood cell types provide crucial immunological functions. Therefore, we assessed five immune-related diseases for which genome-wide association studies previously identified at least 20 loci with a *cis*-eQTL in our meta-analysis. We observed a significant enrichment only for Crohn’s disease (CD), (binomial test, one-tailed P = 0.002, S5 Table): out of 49 unique CD-associated SNPs showing a *cis*-eQTL effect, 11 (22%) were neutrophil-mediated. These 11 SNPs affect the expression of 14 unique genes (ordered by size of interaction effect: *IL18RAP*, *CPEB4*, *RP11-514O12*.*4*, *RNASET2*, *NOD2*, *CISD1*, *LGALS9*, *AC034220*.*3*, *SLC22A4*, *HOTAIRM2*, *ZGPAT*, *LIME1*, *SLC2A4RG*, *and PLCL1*). CD is a chronic inflammatory disease of the intestinal tract. While impaired T-cell responses and defects in antigen presenting cells have been implicated in the pathogenesis of CD, so far little attention has been paid to the role of neutrophils, because its role in the development and maintenance of intestinal inflammation is controversial: homeostatic regulation of the intestine is complex and both a depletion and an increase in neutrophils in the intestinal submucosal space can lead to inflammation. On the one hand, neutrophils are essential in killing microbes that translocate through the mucosal layer. The mucosal layer is affected in CD, but also in monogenic diseases with *neutropeni*a and defects in phagocyte bacterial killing, such as chronic granulomatous disease, glycogen storage disease type I, and congenital neutropenia, leading to various CD phenotypes [23]. On the other hand, an increase in activated neutrophils that secrete pro-inflammatory chemokines and cytokines (including IL18RAP which has a neutrophil specific eQTL) maintains inflammatory responses. Pharmacological interventions for the treatment of CD have been developed to specifically target neutrophils and IL18RAP, including Sagramostim [24] and Natalizumab [25]. These results show clear neutrophil-mediated eQTL effects for various known CD genes, including the archetypal *NOD2* gene. Although CD has previously been shown to have a slightly higher incidence in females [26,27], we did not find any relationship between *NOD2* expression and gender or age (Student's T-test P = 0.08 and Pearson's correlation P = 0.39 respectively; S7 Fig). As such, our results provide deeper insight into the role of neutrophils in CD pathogenesis.

**Fig 4:**
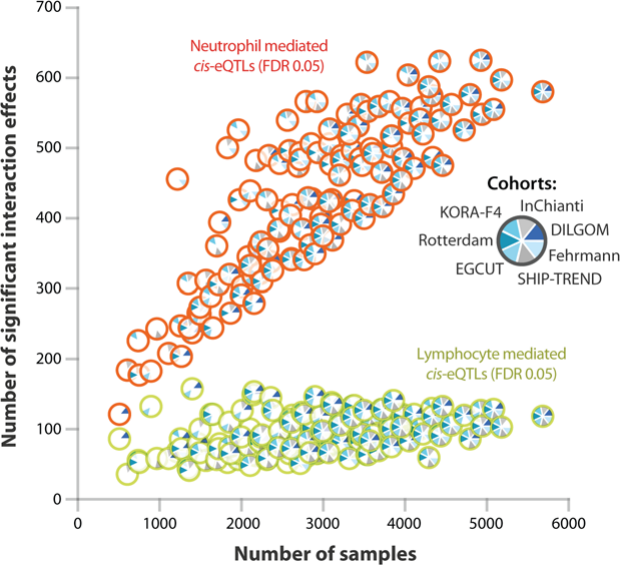
Effect of sample size on power to detect cell type specific *cis*-eQTLs. We systematically excluded datasets from our meta-analysis in order to determine the effect of sample size on our ability to detect significant interaction effects. The number of significant interaction effects was rapidly reduced when the sample size was decreased (the number of unique significant probes given a Bonferroni corrected P-value < 8.1 x 10^–6^ is shown). In general, due to their low abundance in whole blood, lymphoid-mediated *cis*-eQTL effects are harder to detect than neutrophil-mediated cis-eQTL effects.

Large sample sizes are essential in order to find cell-type-mediated *cis*-eQTLs (Fig 4): when we repeat our study on fewer samples (ascertained by systematically excluding more cohorts from our study), the number of significant cell-type-mediated eQTLs decreased rapidly. This was particularly important for the lymphoid-mediated *cis*-eQTLs, because myeloid cells are approximately twice as abundant as lymphoid cells in whole blood. Consequently, detecting lymphoid-mediated *cis*-eQTLs is more challenging than detecting neutrophil-specific *cis*-eQTLs. As whole blood eQTL data is easily collected, we were able to gather a sufficient sample size in order to detect cell-type-mediated or-specific associations without requiring the actual purification of cell types.

## Discussion

Here we have shown that it is possible to infer in which blood cell-types *cis*-eQTLs are operating from a whole blood dataset. Cell-type proportions were predicted and subsequently used in a G x E interaction model. Hundreds of *cis*-eQTLs showed stronger effects in myeloid than lymphoid cell-types and vice versa.

These results were replicated in 6 individual purified cell-type eQTL datasets (two reflecting the myeloid and four reflecting the lymphoid lineage). This indicates our G x E analysis provides important additional biological insights for many SNPs that have previously been found to be associated with complex (molecular) traits.

Here, we concentrated on identifying *cis-*eQTLs that are preferentially operating in either myeloid or lymphoid cell-types. We did not attempt to assess this for specialized cell-types within the myeloid or lymphoid lineage. However, this is possible if cell-counts are available for these cell-types, or if these cell-counts can be predicted by using a proxy for those cell-counts. As such, identification of cell-type mediated eQTLs for previously unstudied cell-types is possible, without the need to generate new data. However, it should be noted that these individual cell-types typically have a rather low abundance within whole blood (e.g. natural killer cells only comprise ~2% of all circulating white blood cells). As a consequence, in order to have sufficient statistical power to identify eQTLs that are mediated by these cell-types, very large whole blood eQTL sample-sizes are required and the cell type of interest should vary in abundance between individuals (analogous to the difference in the number of identified lymphoid mediated *cis*-eQTLs, as compared to the number of neutrophil mediated *cis*-eQTLs, which is likely caused by their difference in abundance in whole blood). For instance we have recently investigated whole peripheral blood RNA-seq data in over 2,000 samples and identified only a handful monocyte specific eQTLs (manuscript in preparation). As such this indicates that in order to identify monocyte specific eQTLs using whole blood, thousands of samples should be studied.

We confined our analyses to a subset of *cis*-eQTLs for which we had previously identified a main effect in whole peripheral blood [22]: for each *cis*-eQTL gene, we only studied the most significantly associated SNP. Considering that for many *cis*-eQTLs multiple, unlinked SNPs exist that independently affect the gene expression levels, it is possible that we have missed myeloid or lymphoid mediation of these secondary *cis*-EQTLs.

The method we have applied to predict the neutrophil percentage in the seven whole blood datasets involves correlation of gene expression probes to cell count abundances and subsequent combination of gene expression probes into a single predictor using PCA. This approach is comparable to other deconvolution methods for methylation and gene expression data [6–11]. However, methods have also been published [28,29] that take a more agnostic approach towards identifying different cell-types and their abundances across different individuals. The Preininger *et al* method [28] identified different axes of gene expression variation in peripheral blood, of which some reflect proxies of certain cell-types. We quantified these axes for each of the samples of the EGCUT and Fehrmann cohorts by creating proxy phenotypes, and subsequently conducted per axis an interaction meta-analysis and indeed identified eQTLs that were significantly mediated by these axes (S6 Table). We first ascertained the Z-scores of the eQTL interaction effects for axis 5 of Preininger *et al*, an axis that is known to correlate strongly with neutrophil percentage. As expected, we observed a very strong correlation with the Z-scores of the eQTL interaction effects for the neutrophil proxy (R = 0.72). While some other axes also mediate eQTLs that might be attributable to differences in other cell-type proportions (e.g. axis 2 that is likely due to differences in reticulocyte proportions), while other axes might even reflect differences in the state in which these cells are (e.g. stimulated immune cells or senescent cells). We believe it is possible that some of these axes might reflect differences in gene activity (rather than differences in cell-type proportions), and that it could be that such differences in gene activity might mediate certain eQTLs. This indicates that by applying novel methods that can summarize gene expression [28,29] and using these summaries as interaction terms when conducting eQTL mapping, various ‘context specific’ eQTLs might become detectable. Although we have shown that the proxy that is created by our method is able to predict neutrophil percentage accurately, this may not be the case for all cell types available in whole blood, which may be greatly dependent upon the ability of individual gene expression probes to differentiate between cell types

However, we anticipate that the (pending) availability of large RNA-seq based eQTL datasets, statistical power to identify cell-type mediated eQTLs using our approach will improve: since RNA-seq enables very accurate gene expression level quantification and is not limited to a set of preselected probes that interrogate well known genes (as is the case for microarrays), the detection of genes that can serve as reliable proxies for individual cell-types will improve. Using RNA-seq data, it is also possible to assess whether SNPs that affect the expression of non-coding transcripts, affect splicing [30] or result in alternative polyadenylation [31] are mediated by specific cell-types.

The method we have used did not account for heteroscedasticity while estimating the standard errors of the interaction term, which may lead to inflated statistics. We therefore compared the standard errors that we used, with standard errors that have been estimated while accounting for heteroscedasticity (using the R-package 'sandwich'). The heteroscedasticity-consistent standard errors and p-values were very similar (Pearson correlation > 0.95; S8 Fig) to the standard errors that did not account for heteroscedasticity. We also note that measurement error in the covariates of the model we have used may cause the inferred betas to be biased. Structural equation modeling may be used to determine unbiased estimates by taking measurement errors of the covariates into account (particularly the neutrophil percentage proxy). However, typically these methods require replicate measurements of the covariates, which were not available for the cohorts in our study. As such, the observed interaction effects may be either underestimated or overestimated, depending on the character and the degree of the measurement error in our covariates.

Although we applied our method to whole blood gene expression data, our method can be applied to any tissue, alleviating the need to sort cells or to perform laser capture micro dissection. The only prerequisite for our method is the availability of a relatively small training dataset with cell count measurements in order to develop a reliable proxy for cell count measurements. Since the number of such training datasets is rapidly increasing and meta-analyses have proven successful [2,22], our approach provides a cost-effective way to identify cell-type-mediated or-specific associations that can supplement results obtained from purified cell type specific datasets, and it is likely to reveal major biological insights.

## Materials and Methods

### Setup of study

This eQTL meta-analysis is based on gene expression intensities measured in whole blood samples. RNA was isolated with either PAXgene Tubes (Becton Dickinson and Co., Franklin Lakes, NJ, USA) or Tempus Tubes (Life Technologies). To measure gene expression levels, Illumina Whole-Genome Expression Beadchips were used (HT12-v3 and HT12-v4 arrays, Illumina Inc., San Diego, USA). Although different identifiers are used across these different platforms, many probe sequences are identical. Meta-analysis could thus be performed if probe-sequences were equal across platforms. Integration of these probe sequences was performed as described before [22]. Genotypes were harmonized using HapMap2-based imputation using the Central European population [32]. In total, the eQTL genotype x environment interaction meta-analysis was performed on seven independent cohorts, comprising a total of 5,863 unrelated individuals (full descriptions of these cohorts can be found in the Supplementary Note). Mix-ups between gene expression samples and genotype samples were corrected using *MixupMapper* [33].

### Gene expression normalization for interaction analysis

Each cohort performed gene expression normalization individually: gene expression data was quantile normalized to the median distribution then log_2_ transformed. The probe and sample means were centered to zero. Gene expression data was then corrected for possible population structure by removing four multi-dimensional scaling components (MDS components obtained from the genotype data using PLINK) using linear regression. Additionally, we corrected for possible confounding factors due to arrays of poor RNA quality. We reasoned that arrays of poor RNA quality generally show expression for genes that are normally lowly expressed within the tissue (e.g. expression for brain genes in whole blood data). As such, the expression profiles for such arrays will deviate overall from arrays with proper RNA quality. To capture such variable arrays, we calculated the first PC from the sample correlation matrix and correlated the first PC with the sample gene expression measurements. Samples with a correlation < 0.9 were removed from further analysis (S9 Fig).

In order to improve statistical power to detect cell-type mediated eQTLs, we corrected the gene expression for technical and batch effects (here we applied principal component analysis and removed per cohort the 40 strongest principal components that affect gene expression). Such procedures are commonly used when conducting *cis-*eQTL mapping [2,5,22,30,31,34]. To minimize the amount of genetic variation removed by this procedure, we performed QTL mapping for each principal component, to ascertain whether genetic variants could be detected that affected the PC. If such an effect was detected, we did not correct the gene expression data for that particular PC [22]. As a result, this procedure also removed the majority of the variation that explained the correlation between neutrophil percentage and gene expression (S10 Fig), minimizing issues with possible collinearity when testing the interaction effects. We chose to remove 40 PCs based on our previous study results, which suggested that this was the optimum for detecting eQTLs [22]. We would like to stress that while PC-corrected gene expression data was then used as the outcome variable in our gene x environment interaction model, we used gene expression data that was not corrected for PCs to initially create the neutrophil cell percentage proxy.

### Creating a proxy for neutrophil cell percentage from quantile normalized and log_2_ transformed gene expression data

To be able to determine whether a *cis*-eQTL is mediated by neutrophils, we reasoned that such a *cis*-eQTL would show a larger effect size in individuals with a higher percentage of neutrophils than in individuals with a lower percentage. However, this required the percentage of neutrophils in whole blood to be known, and cell-type percentage measurements were not available for all of the cohorts. We therefore created a proxy phenotype that reflected the actual neutrophil percentage that would also be applicable to datasets without neutrophil percentage measurements. In the EGCUT dataset, we first quantile normalized and log_2_ transformed the raw expression data. We then correlated the gene expression levels of individual probes with the neutrophil percentage, and selected 58 gene expression probes showing a high positive correlation (Spearman R > 0.57). Here, we chose to use the quantile normalized, log_2_ transformed gene expression data that was not corrected for principal components, since correction for principal components would remove the correlation structure between gene expression and neutrophil percentage (S10 Fig).

In each independent cohort, we corrected for possible confounding factors due to arrays with poor RNA quality, by correlating the quantile normalized and log_2_ transformed gene expression measurements against the first PC determined from the sample correlation matrix. Only samples with a high correlation (r ≥ 0.9) were included in further analyses. Then, for each cohort, we calculated a correlation matrix for the neutrophil proxy probes (the probes selected from the EGCUT cohort). The gene expression data used was quantile normalized, log_2_ transformed and corrected for MDS components. Applying PCA to the correlation matrix, we then obtained PCs that described the variation among the probes selected from the EGCUT cohort. As the first PC (PC1) contributes the largest amount of variation, we considered PC1 as a proxy-phenotype for the cell type percentages.

### Determining cell-type mediation using an interaction model

Considering the overlap between the cohorts in this study and our previous study, we limited our analysis to the 13,124 *cis*-eQTLs having a significant effect (false discovery rate, FDR < 0.05) in our previous study [22]. This included 8,228 unique Illumina HT12v3 probes and 10,260 unique SNPs (7,674 SNPs that showed the strongest effect per probe, and 2,586 SNPs previously associated with complex traits and diseases, as reported in the Catalog of Published Genome-Wide Association Studies [1], on 23^rd^ September, 2013).

We defined the model for single marker *cis*-eQTL mapping as follows:

$$Y\approx I+\beta_{1}*G+e$$

where **Y** is the gene expression of the gene, **β**_**1**_ is the slope of the linear model, **G** is the genotype, **I** is the intercept with the y-axis, and **e** is the general error term for any residual variation not explained by the rest of the model.

We then extended the typical linear model for single marker *cis*-eQTL mapping to include a covariate as an independent variable, and captured the interaction between the genotype and the covariate using an interaction term:

$$Y\approx I+\beta_{1}*G+\beta_{2}*P+\beta_{3}*P:G+e$$

where P (cell-type proxy) is the covariate, and P:G is the interaction term between the covariate and the genotype. We used gene expression data corrected for 40 PCs as the predicted variable (**Y**). The interaction terms were then meta-analyzed over all cohorts using a Z-score method, weighted for the sample size [35].

### Multiple testing correction

Since the gene-expression data has a correlated structure (i.e. co-expressed genes) and the genotype data also has a correlated structure (i.e. linkage disequilibrium between SNPs), a Bonferroni correction would be overly stringent. We therefore first estimated the effective number of uncorrelated tests by using permuted eQTL results from our previous *cis-*eQTL meta-analysis [22]. The most significant P-value in these permutations was 8.15 x 10^–5^, when averaged over all permutations. As such, the number of effective tests = 0.5 / 8.15 x 10^–5^ ≈ 6134, which is approximately half the number of correlated *cis*-eQTL tests that we conducted (= 13,124). Next, we controlled the FDR at 0.05 for the interaction analysis: for a given P-value threshold in our interaction analysis, we calculated the number of expected results (given the number of effective tests and a uniform distribution) and determined the observed number of eQTLs that were below the given P-value threshold (FDR = number of expected p-values below threshold / number of observed p-values below threshold). At an FDR of 0.05, our nominal p-value threshold was 0.009 (corresponding to an absolute interaction effect Z-score of 2.61).

### Cell-type specific *cis*-eQTLs and disease

For each trait in the GWAS catalog, we pruned all SNPs with a GWAS association P-value below 5 x 10^–8^, using an r^2^ threshold of 0.2. We only considered traits that had more than 20 significant eQTL SNPs after pruning (irrespective of cell-type mediation). Then, we determined the proportion of pruned neutrophil-mediated *cis*-eQTLs for the trait relative to all the neutrophil-mediated *cis*-eQTLs. The difference between both proportions was then tested using a binomial test.

### Data access

The source code and documentation for this type of analysis are available as part of the eQTL meta-analysis pipeline at https://github.com/molgenis/systemsgenetics

Summary results are available from http://www.genenetwork.nl/celltype

### Accession numbers

Discovery cohorts: Fehrmann (GSE 20142), SHIP-TREND (GSE 36382), Rotterdam Study (GSE 33828), EGCUT (GSE 48348), DILGOM (E-TABM-1036), InCHIANTI (GSE 48152), KORA F4 (E-MTAB-1708). Replication Cohorts: Stranger (E-MTAB-264), Oxford (E-MTAB-945).

## Supporting Information

S1 Fig Neutrophil percentage and gene expression correlation distribution in EGCUT. In order to predict the neutrophil percentage, we selected 58 gene expression probes (0.1% of the dataset) that strongly positively correlated with neutrophil percentage in the EGCUT dataset (Spearman R > 0.57, P < 3 x 10^–72^, n = 825).
(TIF)

S2 Fig Comparison of expression in the BLUEPRINT study. The 58 probes we used to estimate neutrophil percentage map to 44 unique genes. We compared the gene expression levels for these genes using RNA-seq data from the BLUEPRINT consortium among 14 different cell types. For most of these cell-types multiple biological replicates have been assayed. We quantile normalized, log_2_ transformed and then centered the expression levels for every individual gene to a mean of zero and a standard deviation of one. We observed that 42 of these 44 genes show significantly higher expression (Student's T-test P < 0.001), as compared to the other 13 cell types. NS: non significant.
(TIF)

S3 Fig Relationship between neutrophil percentage, age and gender. We correlated the actual neutrophil percentage (top) and the inferred neutrophil percentage (bottom) with age in the EGCUT dataset (n = 825) and observed that there is a low, but significant correlation between age and neutrophil percentage. However, neutrophil percentage is not significantly associated with gender.
(TIF)

S4 Fig Stability of neutrophil percentage prediction. We tested the stability of our neutrophil percentage prediction in the EGCUT dataset (n = 825). From the list of 100 probes showing highest correlation with neutrophil percentage, we randomly selected a number of probes (increments of 5 probes, 1000 permutations per increment) and repeated the neutrophil percentage prediction. When including > 10 probes, the neutrophil prediction displays stable correlation with the actual neutrophil percentage (Spearman R ~0.75) and near perfect correlation with the predicted neutrophil percentage used in the meta-analysis (Spearman R ~0.99). Error bars denote standard deviation. Red line denotes the number of gene expression probes the different cohorts in this study used to estimate neutrophil percentage.
(TIF)

S5 Fig *Cis*-eQTL effect size and cell type specificity. 71% of the cis-eQTLs that were identified as being cell type specific by our method show an effect size larger than 0.03 in our original cis-eQTL meta-analysis (Westra et al, 2013), compared to 21% for those that do not have a significant interaction effect.
(TIF)

S6 Fig Comparison of effect sizes and effect direction between datasets. Comparison of interaction effect Z-scores shows a high consistent direction of effect between datasets and with the meta-analysis for those interaction effects significant at FDR < 0.05.
(TIF)

S7 Fig Relationship between *NOD2* gene expression levels, age and gender. We correlated the actual *NOD2* gene expression levels with age in the EGCUT dataset (n = 825, normalized using log_2_ transformed and quantile normalization, and gene expression levels corrected for 40 principal components) and observed that there is a low, but significant correlation between age and *NOD2* gene expression in the log_2_ transformed and quantile normalized data (top), which becomes insignificant when correcting the gene expression data for 40 principal components (which was used to determine the neutrophil interaction effect; bottom). However, *NOD2* gene expression levels are not significantly associated with gender.
(TIF)

S8 Fig Effect of robust estimation of standard errors. The interaction model we used does not take heteroscedasticity into account. Therefore, we determined standard errors using the 'sandwich' package in R, which allows for the estimation of robust standard errors. We observed strong correlation between standard errors, Z-scores and p-values by our model and a model that applies robust estimation of standard errors in the EGCUT (top) and Fehrmann datasets (bottom).
(TIF)

S9 Fig Principal components on gene expression data. Principal component 1 (PC1) and principal component 2 per study. Samples with a correlation < 0.9 with PC1 (red) were excluded from analysis.
(TIF)

S10 Fig Neutrophil percentage and principal component correction. The gene expression data that was used for the interaction meta-analysis was corrected for up to 40 principal components. In order to retain genetic variation in the gene expression data, components that showed a significant correlation with genotypes were not removed. In the EGCUT dataset (n = 825), many of these components also strongly correlate with neutrophil percentage (top) and inferred neutrophil percentage (bottom). The majority of the variation in gene expression explained by these components (right) was however removed from this dataset.
(TIF)

S1 Table List of 58 Illumina HT12v3 probes used for calculating the estimated neutrophil percentage principal component score and their correlation with neutrophil percentage in the EGCUT dataset (n = 825). (XLSX)

S2 Table Summary statistics for the interaction analysis. (XLSX)

S3 Table Results of the interaction analysis. (XLSX)

S4 Table Summary statistics showing the effect size (correlation coefficient) in each of the tested replication datasets. (XLSX)

S5 Table Results of the neutrophil mediated cis-eQTL disease enrichment analysis. (XLSX)

S6 Table We created proxy phenotypes for the 9 axes of variation described by Preininger et al [28] within the EGCUT (n = 891) and Fehrmann (n = 1,220) cohorts. We then meta-analyzed the interaction terms for these two cohorts and observed that several axes mediate eQTL effects. The Z-scores for the interaction effects for axis 5 correlate strongly with the Z-scores for interaction effects for the neutrophil proxy (R = 0.74).
(XLSX)

S1 Text Supplementary note. (DOCX)

## References

[1] Hindorff LA, Sethupathy P, Junkins H a, Ramos EM, Mehta JP, et al (2009) Potential etiologic and functional implications of genome-wide association loci for human diseases and traits. Proc Natl Acad Sci U S A 106: 9362–9367. 10.1073/pnas.0903103106 19474294 PMC2687147

[2] Fehrmann RSN, Jansen RC, Veldink JH, Westra H-J, Arends D, et al (2011) Trans-eQTLs Reveal That Independent Genetic Variants Associated with a Complex Phenotype Converge on Intermediate Genes, with a Major Role for the HLA. PLoS Genet 7: 14. 10.1371/journal.pgen.1002197 PMC3150446 21829388

[3] Brown CD, Mangravite LM, Engelhardt BE (2013) Integrative Modeling of eQTLs and Cis-Regulatory Elements Suggests Mechanisms Underlying Cell Type Specificity of eQTLs. PLoS Genet 9: e1003649 10.1371/journal.pgen.1003649 23935528 PMC3731231

[4] Fairfax BPB, Makino S, Radhakrishnan J, Plant K, Leslie S, et al (2012) Genetics of gene expression in primary immune cells identifies cell-specific master regulators and roles of HLA alleles. Nat Genet 44: 502–510. 10.1038/ng.2205.GENETICS 22446964 PMC3437404

[5] Fu J, Wolfs MGM, Deelen P, Westra H-J, Fehrmann RSN, et al (2012) Unraveling the regulatory mechanisms underlying tissue-dependent genetic variation of gene expression. PLoS Genet 8: e1002431 10.1371/journal.pgen.1002431 22275870 PMC3261927

[6] Houseman EA, Accomando WP, Koestler DC, Christensen BC, Marsit CJ, et al (2012) DNA methylation arrays as surrogate measures of cell mixture distribution. BMC Bioinformatics 13: 86 10.1186/1471-2105-13-86 22568884 PMC3532182

[7] Houseman EA, Molitor J, Marsit CJ (2014) Reference-free cell mixture adjustments in analysis of DNA methylation data. Bioinformatics. 10.1093/bioinformatics/btu029 PMC4016702 24451622

[8] Accomando WP, Wiencke JK, Houseman EA, Nelson HH, Kelsey KT (2014) Quantitative reconstruction of leukocyte subsets using DNA methylation. Genome Biol 15: R50 10.1186/gb-2014-15-3-r50 24598480 PMC4053693

[9] Jaffe AE, Irizarry RA (2014) Accounting for cellular heterogeneity is critical in epigenome-wide association studies. Genome Biol 15: R31 10.1186/gb-2014-15-2-r31 24495553 PMC4053810

[10] Leek JT, Storey JD (2007) Capturing heterogeneity in gene expression studies by surrogate variable analysis. PLoS Genet 3: 1724–1735. 17907809 10.1371/journal.pgen.0030161 PMC1994707

[11] Shen-Orr SS, Tibshirani R, Khatri P, Bodian DL, Staedtler F, et al (2010) Cell type-specific gene expression differences in complex tissues. Nat Methods 7: 287–289. 10.1038/nmeth.1439 20208531 PMC3699332

[12] Metspalu A (2004) The Estonian Genome Project. Drug Dev Res 62: 97–101. 10.1002/ddr.10371

[13] Tanaka T, Shen J, Abecasis GR, Kisialiou A, Ordovas JM, et al (2009) Genome-wide association study of plasma polyunsaturated fatty acids in the InCHIANTI Study. PLoS Genet 5: e1000338 10.1371/journal.pgen.1000338 19148276 PMC2613033

[14] Hofman A, Darwish Murad S, van Duijn CM, Franco OH, Goedegebure A, et al (2013) The Rotterdam Study: 2014 objectives and design update. Eur J Epidemiol 28: 889–926. 10.1007/s10654-013-9866-z 24258680

[15] Völzke H, Alte D, Schmidt CO, Radke D, Lorbeer R, et al (2011) Cohort profile: the study of health in Pomerania. Int J Epidemiol 40: 294–307. 10.1093/ije/dyp394 20167617

[16] Mehta D, Heim K, Herder C, Carstensen M, Eckstein G, et al (2013) Impact of common regulatory single-nucleotide variants on gene expression profiles in whole blood. Eur J Hum Genet 21: 48–54. 10.1038/ejhg.2012.106 22692066 PMC3522194

[17] Inouye M, Silander K, Hamalainen E, Salomaa V, Harald K, et al (2010) An immune response network associated with blood lipid levels. PLoS Genet 6: e1001113 10.1371/journal.pgen.1001113 20844574 PMC2936545

[18] Nalls MA, Couper DJ, Tanaka T, van Rooij FJA, Chen M-H, et al (2011) Multiple loci are associated with white blood cell phenotypes. PLoS Genet 7: e1002113 10.1371/journal.pgen.1002113 21738480 PMC3128114

[19] Crosslin DR, McDavid A, Weston N, Nelson SC, Zheng X, et al (2012) Genetic variants associated with the white blood cell count in 13,923 subjects in the eMERGE Network. Hum Genet 131: 639–652. 10.1007/s00439-011-1103-9 22037903 PMC3640990

[20] Grisham MB, Engerson TD, McCord JM, Jones HP (1985) A comparative study of neutrophil purification and function. J Immunol Methods 82: 315–320. 2995497 10.1016/0022-1759(85)90363-1

[21] Blueprint DCC Portal (n.d.). Available: http://dcc.blueprint-epigenome.eu/#/home. Accessed 25 November 2014.

[22] Westra H-J, Peters MJ, Esko T, Yaghootkar H, Schurmann C, et al. (2013) Systematic identification of trans eQTLs as putative drivers of known disease associations. Nat Genet. 10.1038/ng.2756 PMC3991562 24013639

[23] Uhlig HH (2013) Monogenic diseases associated with intestinal inflammation: implications for the understanding of inflammatory bowel disease. Gut 62: 1795–1805. 10.1136/gutjnl-2012-303956 24203055

[24] Korzenik JR, Dieckgraefe BK, Valentine JF, Hausman DF, Gilbert MJ (2005) Sargramostim for active Crohn’s disease. N Engl J Med 352: 2193–2201. 15917384 10.1056/NEJMoa041109

[25] Ghosh S, Goldin E, Gordon FH, Malchow HA, Rask-Madsen J, et al (2003) Natalizumab for active Crohn’s disease. N Engl J Med 348: 24–32. 12510039 10.1056/NEJMoa020732

[26] Montgomery SM, Wakefield AJ, Ekbom A (2003) Sex-Specific Risks for Pediatric Onset Among Patients With Crohn’s Disease. Clin Gastroenterol Hepatol 1: 303–309. 10.1016/S1542-3565(03)00135-6 15017672

[27] Goodman WA, Garg RR, Reuter BK, Mattioli B, Rissman EF, et al (2014) Loss of estrogen-mediated immunoprotection underlies female gender bias in experimental Crohn’s-like ileitis. Mucosal Immunol 7: 1255–1265. 10.1038/mi.2014.15 24621993 PMC4139459

[28] Preininger M, Arafat D, Kim J, Nath AP, Idaghdour Y, et al (2013) Blood-informative transcripts define nine common axes of peripheral blood gene expression. PLoS Genet 9: e1003362 10.1371/journal.pgen.1003362 23516379 PMC3597511

[29] Fehrmann RSN, Karjalainen JM, Krajewska M, Westra H-J, Maloney D, et al (2015) Gene expression analysis identifies global gene dosage sensitivity in cancer. Nat Genet 47: 115–125. 10.1038/ng.3173 25581432

[30] Lappalainen T, Sammeth M, Friedländer MR, ‘t Hoen PAC, Monlong J, et al (2013) Transcriptome and genome sequencing uncovers functional variation in humans. Nature 501: 506–511. 10.1038/nature12531 24037378 PMC3918453

[31] Zhernakova D V, de Klerk E, Westra H-J, Mastrokolias A, Amini S, et al (2013) DeepSAGE reveals genetic variants associated with alternative polyadenylation and expression of coding and non-coding transcripts. PLoS Genet 9: e1003594 10.1371/journal.pgen.1003594 23818875 PMC3688553

[32] The International HapMap Consortium (2003) The International HapMap Project. 426: 789–796. 14685227 10.1038/nature02168

[33] Westra H-J, Jansen RC, Fehrmann RSN, te Meerman GJ, van Heel D, et al (2011) MixupMapper: correcting sample mix-ups in genome-wide datasets increases power to detect small genetic effects. Bioinformatics 27: 2104–2111. 10.1093/bioinformatics/btr323 21653519

[34] Dubois PCA, Trynka G, Franke L, Hunt KA, Romanos J, et al (2010) Multiple common variants for celiac disease influencing immune gene expression. Nat Genet 42: 295–302. 10.1038/ng.543 20190752 PMC2847618

[35] Whitlock MC (2005) Combining probability from independent tests: the weighted Z-method is superior to Fisher’s approach. J Evol Biol 18: 1368–1373. 10.1111/j.1420-9101.2005.00917.x 16135132

