# Supplementary figures and images for: Cell Specific eQTL Analysis without Sorting Cells
Source: PLoS Genet. 2015 May 8;11(5):e1005223. doi: 10.1371/journal.pgen.1005223 (PMC4425538; doi:10.1371/journal.pgen.1005223)

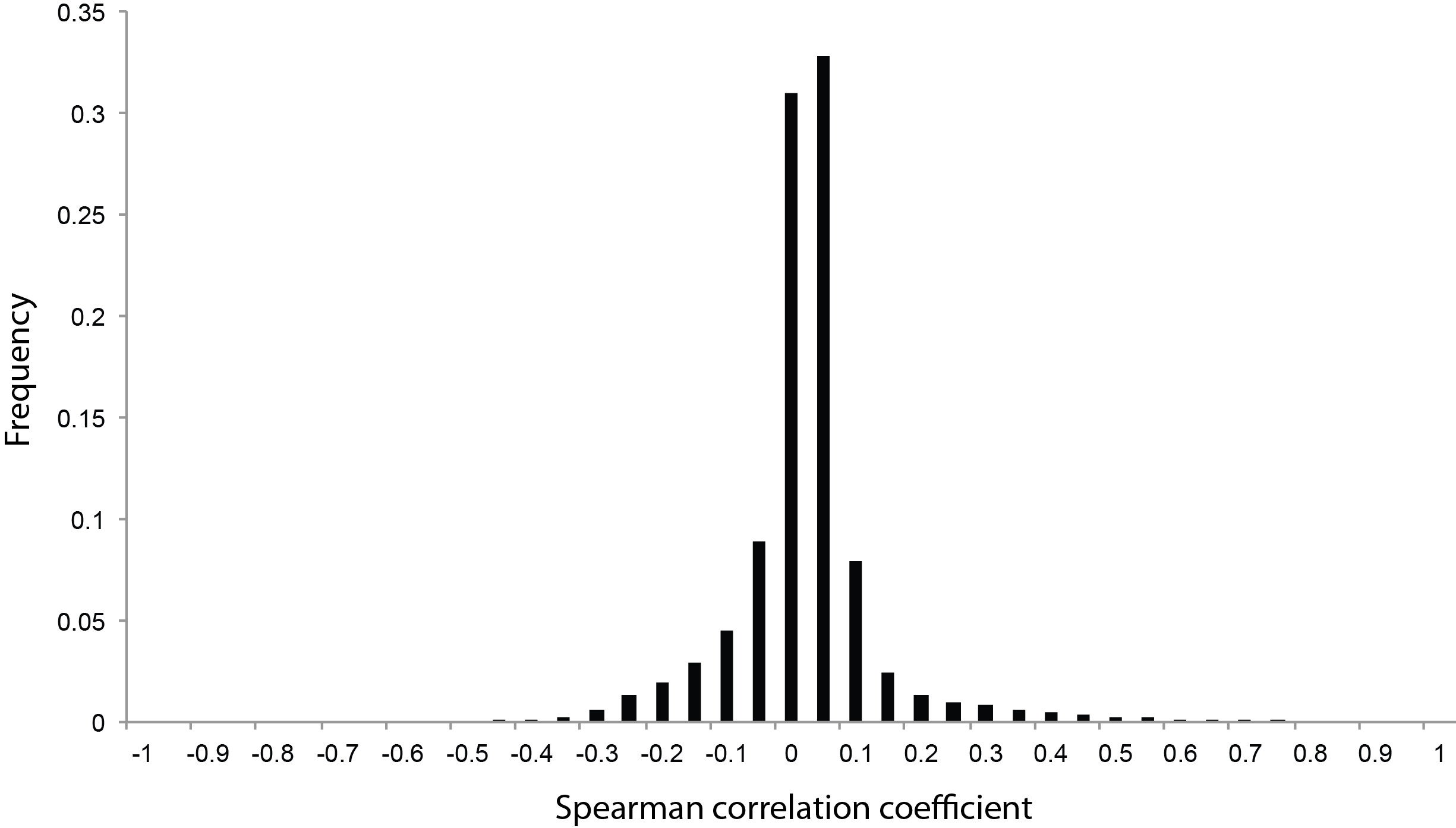

Supplement: S1 Fig — In order to predict the neutrophil percentage, we selected 58 gene expression probes (0.1% of the dataset) that strongly positively correlated with neutrophil percentage in the EGCUT dataset (Spearman R > 0.57, P < 3 x 10^–72^, n = 825). (TIF) [file pgen.1005223.s001.tif]

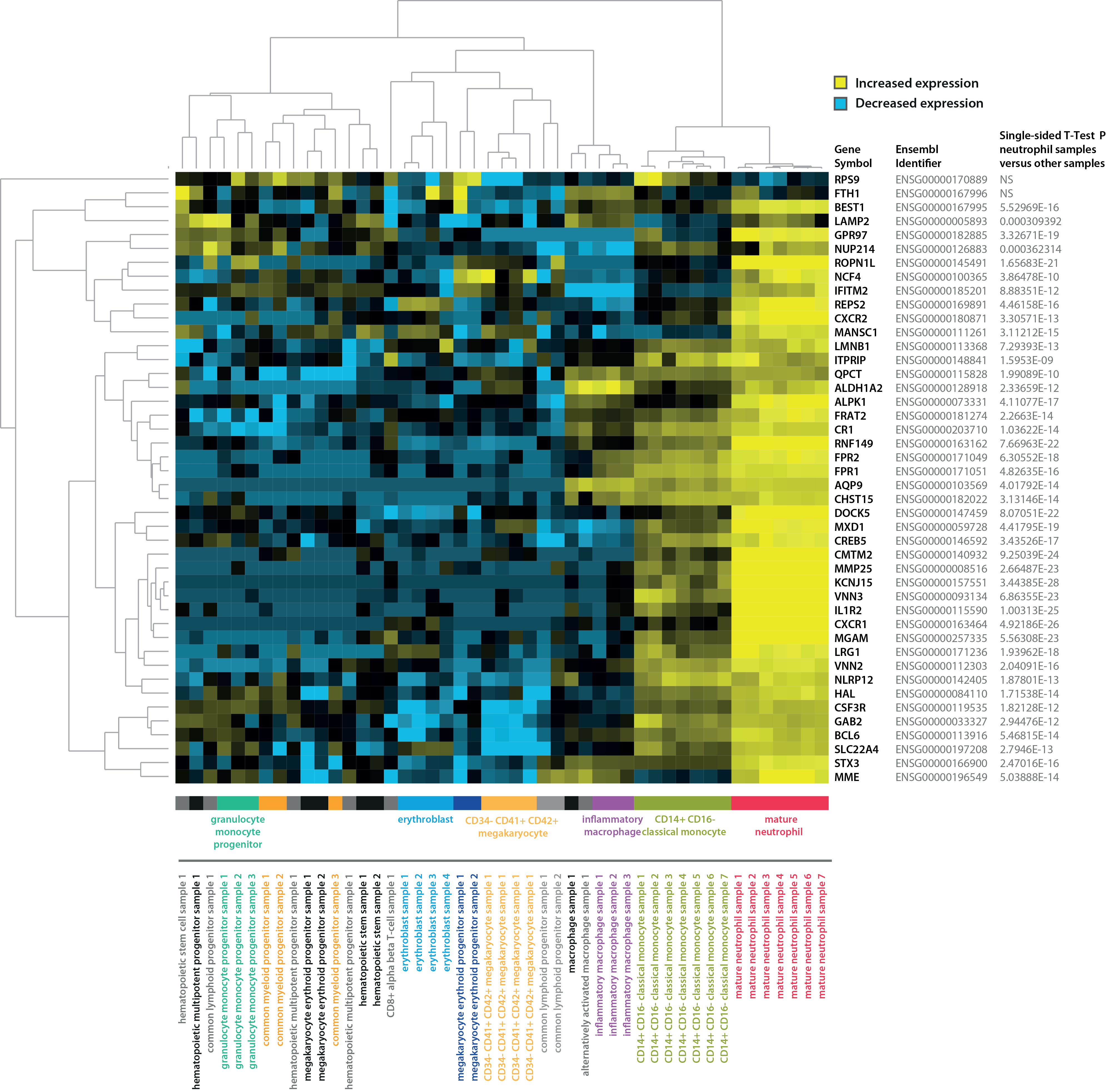

Supplement: S2 Fig — The 58 probes we used to estimate neutrophil percentage map to 44 unique genes. We compared the gene expression levels for these genes using RNA-seq data from the BLUEPRINT consortium among 14 different cell types. For most of these cell-types multiple biological replicates have been assayed. We quantile normalized, log2 transformed and then centered the expression levels for every individual gene to a mean of zero and a standard deviation of one. We observed that 42 of these 44 genes show significantly higher expression (Student's T-test P < 0.001), as compared to the other 13 cell types. NS: non significant. (TIF) [file pgen.1005223.s002.tif]

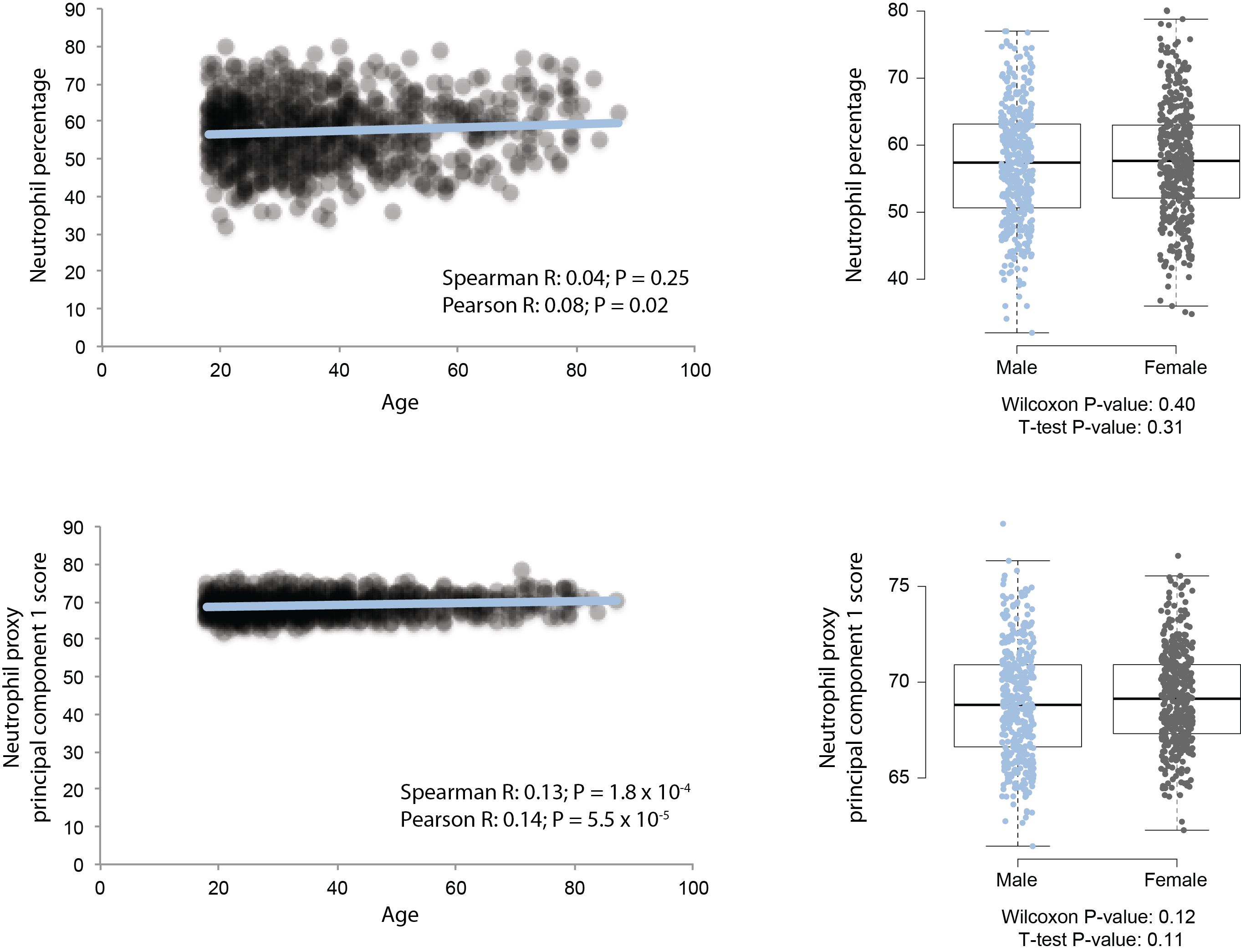

Supplement: S3 Fig — We correlated the actual neutrophil percentage (top) and the inferred neutrophil percentage (bottom) with age in the EGCUT dataset (n = 825) and observed that there is a low, but significant correlation between age and neutrophil percentage. However, neutrophil percentage is not significantly associated with gender. (TIF) [file pgen.1005223.s003.tif]

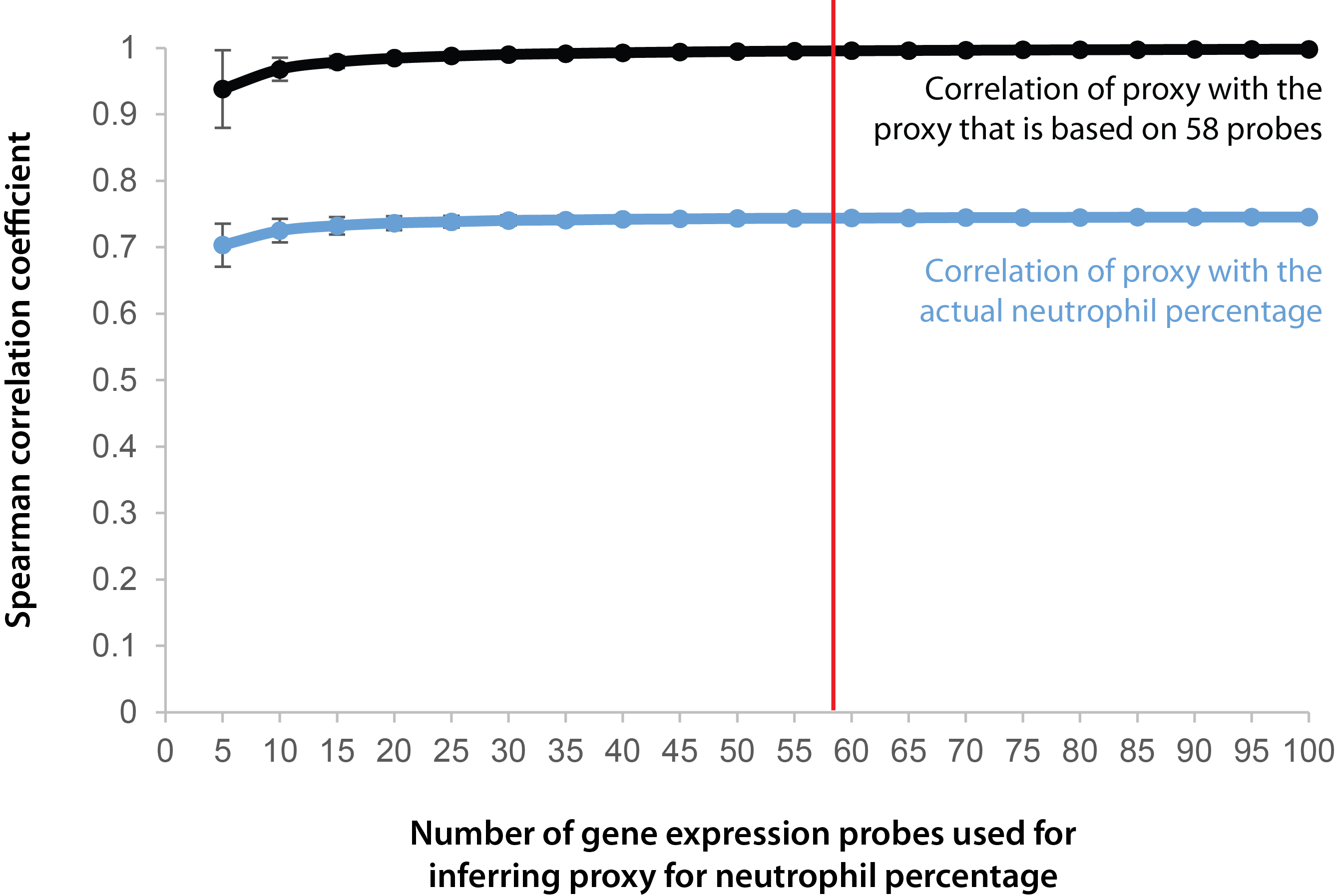

Supplement: S4 Fig — We tested the stability of our neutrophil percentage prediction in the EGCUT dataset (n = 825). From the list of 100 probes showing highest correlation with neutrophil percentage, we randomly selected a number of probes (increments of 5 probes, 1000 permutations per increment) and repeated the neutrophil percentage prediction. When including > 10 probes, the neutrophil prediction displays stable correlation with the actual neutrophil percentage (Spearman R ~0.75) and near perfect correlation with the predicted neutrophil percentage used in the meta-analysis (Spearman R ~0.99). Error bars denote standard deviation. Red line denotes the number of gene expression probes the different cohorts in this study used to estimate neutrophil percentage. (TIF) [file pgen.1005223.s004.tif]

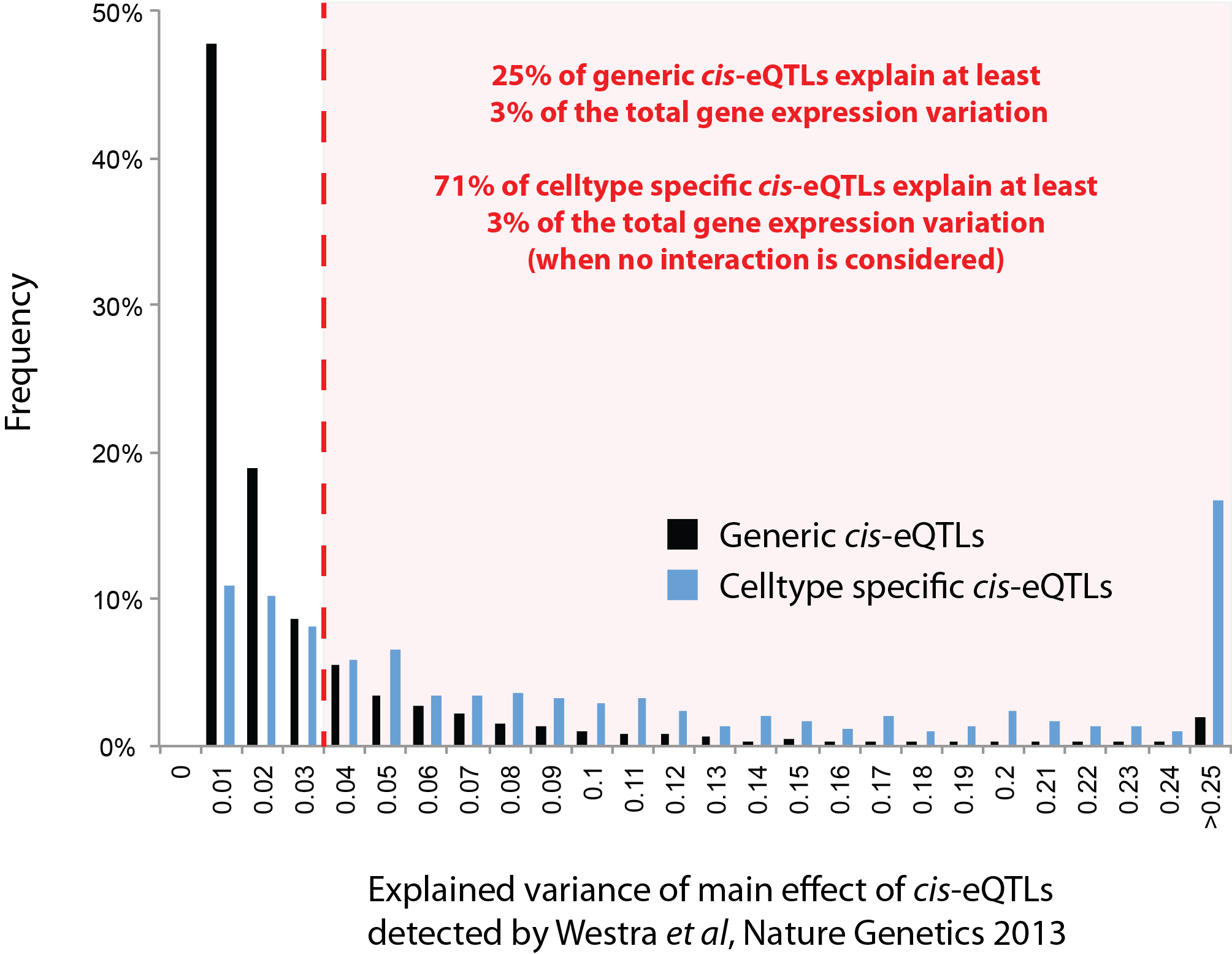

Supplement: S5 Fig — 71% of the cis-eQTLs that were identified as being cell type specific by our method show an effect size larger than 0.03 in our original cis-eQTL meta-analysis (Westra et al, 2013), compared to 21% for those that do not have a significant interaction effect. (TIF) [file pgen.1005223.s005.tif]

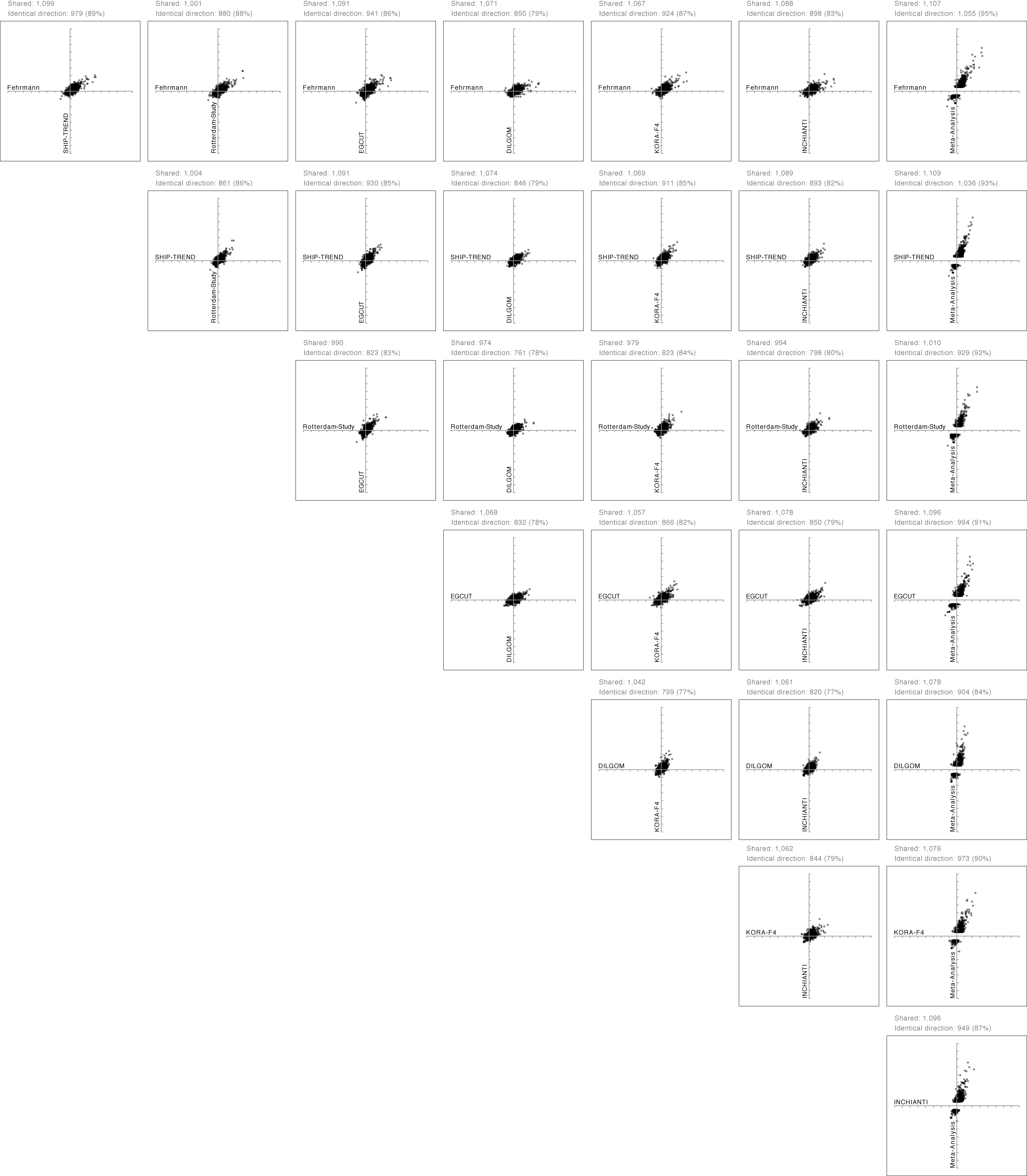

Supplement: S6 Fig — Comparison of interaction effect Z-scores shows a high consistent direction of effect between datasets and with the meta-analysis for those interaction effects significant at FDR < 0.05. (TIF) [file pgen.1005223.s006.tif]

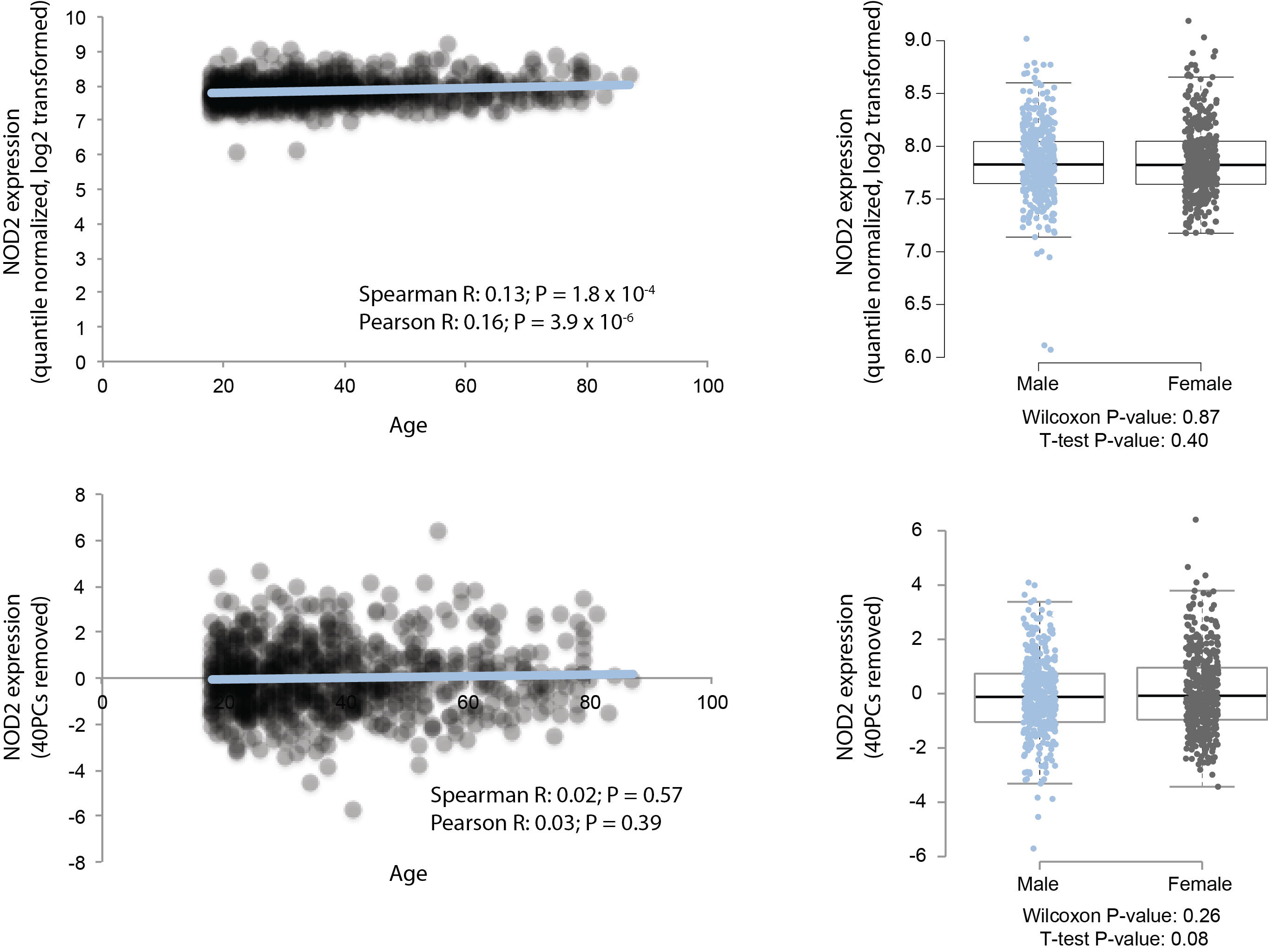

Supplement: S7 Fig — We correlated the actual NOD2 gene expression levels with age in the EGCUT dataset (n = 825, normalized using log2 transformed and quantile normalization, and gene expression levels corrected for 40 principal components) and observed that there is a low, but significant correlation between age and NOD2 gene expression in the log2 transformed and quantile normalized data (top), which becomes insignificant when correcting the gene expression data for 40 principal components (which was used to determine the neutrophil interaction effect; bottom). However, NOD2 gene expression levels are not significantly associated with gender. (TIF) [file pgen.1005223.s007.tif]

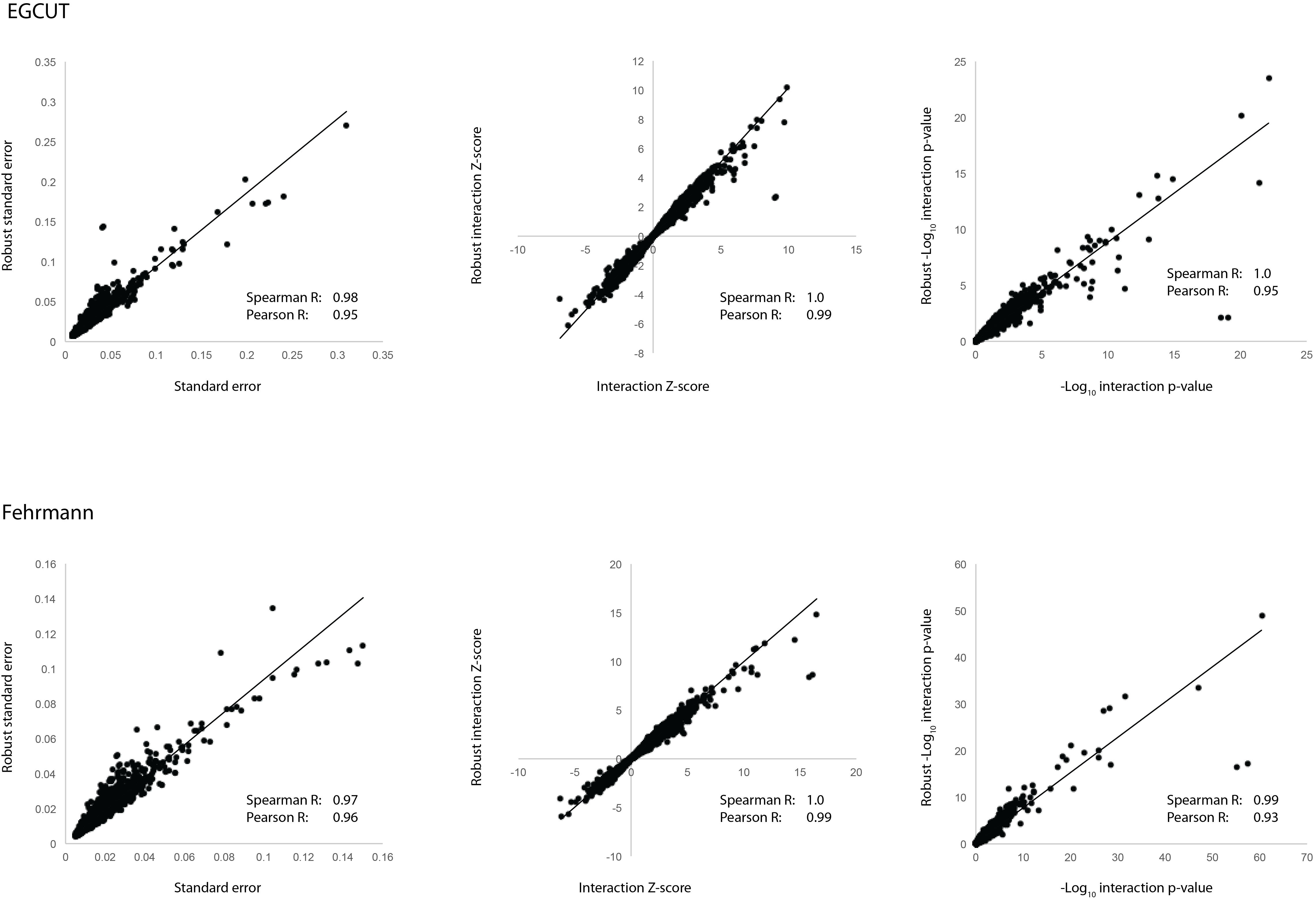

Supplement: S8 Fig — The interaction model we used does not take heteroscedasticity into account. Therefore, we determined standard errors using the 'sandwich' package in R, which allows for the estimation of robust standard errors. We observed strong correlation between standard errors, Z-scores and p-values by our model and a model that applies robust estimation of standard errors in the EGCUT (top) and Fehrmann datasets (bottom). (TIF) [file pgen.1005223.s008.tif]

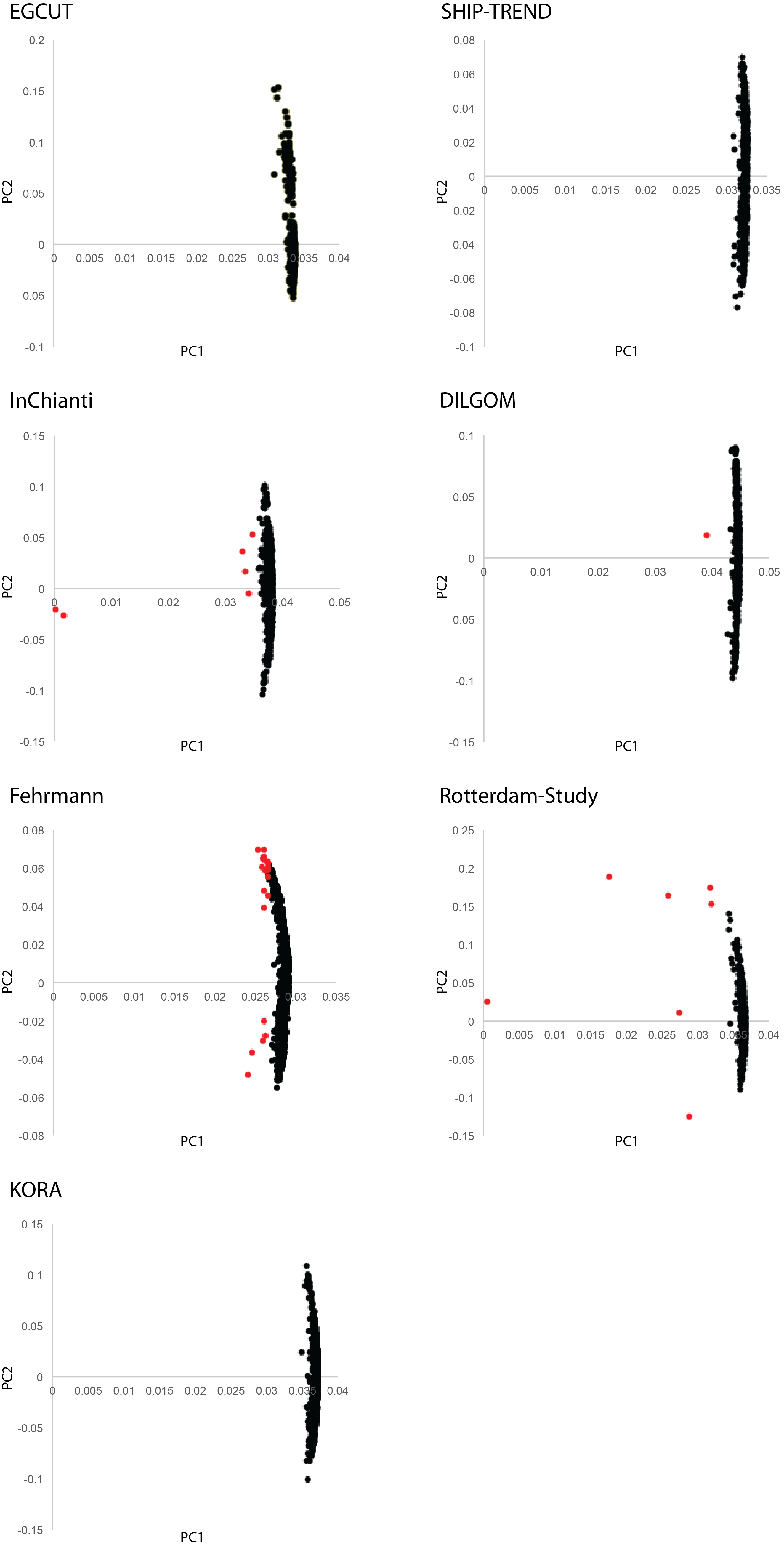

Supplement: S9 Fig — Principal component 1 (PC1) and principal component 2 per study. Samples with a correlation < 0.9 with PC1 (red) were excluded from analysis. (TIF) [file pgen.1005223.s009.tif]

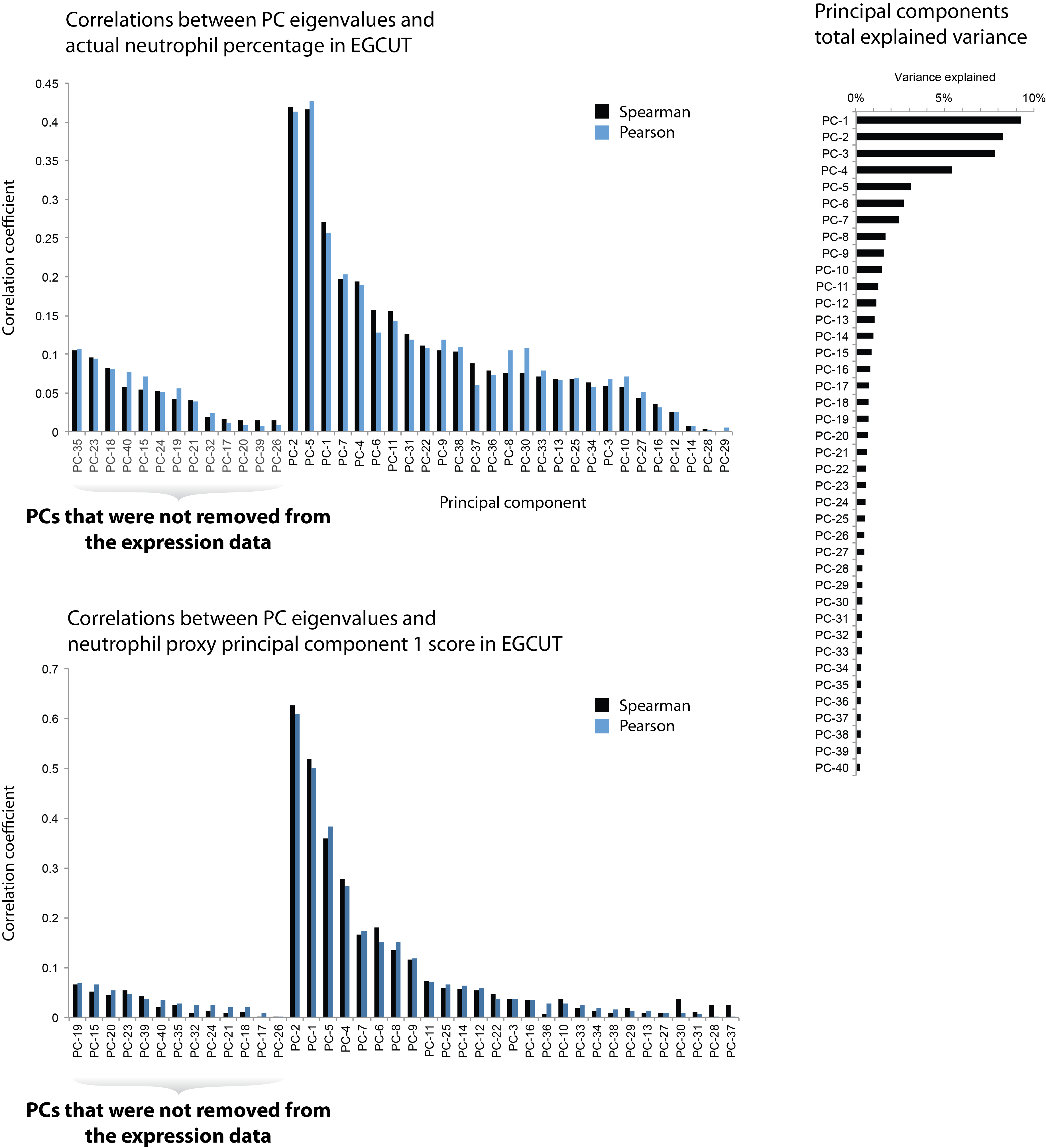

Supplement: S10 Fig — The gene expression data that was used for the interaction meta-analysis was corrected for up to 40 principal components. In order to retain genetic variation in the gene expression data, components that showed a significant correlation with genotypes were not removed. In the EGCUT dataset (n = 825), many of these components also strongly correlate with neutrophil percentage (top) and inferred neutrophil percentage (bottom). The majority of the variation in gene expression explained by these components (right) was however removed from this dataset. (TIF) [file pgen.1005223.s010.tif]
