# Supplementary material for: Cell Specific eQTL Analysis without Sorting Cells
Source: PLoS Genet. 2015 May 8;11(5):e1005223. doi: 10.1371/journal.pgen.1005223 (PMC4425538; doi:10.1371/journal.pgen.1005223)
Supplement: S1 Text — (DOCX) [file pgen.1005223.s017.docx]

Cell-type specific eQTL analysis without the need to sort cells

Harm-Jan Westra^1,47^, Danny Arends^2,47^, Tõnu Esko^3,4,5,6^, Marjolein J. Peters^7,8^, Claudia Schurmann^9,10^, Katharina Schramm^11,12^, Johannes Kettunen^13,14,15^, Hanieh Yaghootkar^16^, Benjamin P. Fairfax^17,18^, Anand Kumar Andiappan^19^, Yang Li^1,2^, Jingyuan Fu^1^, Juha Karjalainen^1^, Mathieu Platteel^1^, Marijn Visschedijk^1,20^, Rinse K Weersma^20^, Silva Kasela^3,21^, Lili Milani^3^, Liina Tserel^22^, Pärt Peterson^22^, Eva Reinmaa^3^, Albert Hofman^8,23^, André G. Uitterlinden^7,8,23^, Fernando Rivadeneira^7,8,23^, Georg Homuth^9^, Astrid Petersmann^24^, Roberto Lorbeer^25^, Holger Prokisch^26,27^, Thomas Meitinger^26,27,28,29^, Christian Herder^30,31^, Michael Roden^30,31,32^, Harald Grallert^33^, Samuli Ripatti^34,35,36,37^, Markus Perola^3,15^, Andrew R Wood^16^, David Melzer^38^, Luigi Ferrucci^39^, Andrew B Singleton^40^, Dena G. Hernandez^40,41^, Julian C. Knight^17^, Rossella Melchiotti^19,42^, Bernett Lee^19^, Michael Poidinger^19^, Francesca Zolezzi^19^, Anis Larbi^19^, De Yun Wang^43^, Leonard H. van den Berg^44^, Jan H. Veldink^44^, Olaf Rotzschke^19^, Seiko Makino^17^, Veikko Salomaa^15^, Konstantin Strauch^45,46^, Uwe Völker^9^, Joyce B.J. van Meurs^7,8^, Andres Metspalu^3^, Cisca Wijmenga^1^, Ritsert C. Jansen^2,48^, Lude Franke^1,48,49^

1 University of Groningen, University Medical Center Groningen, Department of Genetics, Hanzeplein 1, 9700RB, Groningen, The Netherlands. 2 Groningen Bioinformatics Centre, University of Groningen, P.O. Box 11103, 9700 CC Groningen, The Netherlands. 3 Estonian Genome Center, University of Tartu, Riia 23b, 51010 Tartu, Estonia. 4 Divisions of Endocrinology, Boston Children's Hospital, Boston, 02115, USA. 5 Department of Genetics, Harvard Medical School, Boston, 02115, USA. 6 Broad Institute, Cambridge, 02142, USA. 7 Department of Internal Medicine, Erasmus Medical Centre Rotterdam, the Netherlands. 8 The Netherlands Genomics Initiative-sponsored Netherlands Consortium for Healthy Aging (NGI-NCHA), Leiden / Rotterdam, the Netherlands. 9 Interfaculty Institute of Genetics and Functional Genomics, University Medicine Greifswald, Friedrich-Ludwig-Jahn-Str. 15A, 17475 Greifswald, Germany. 10 The Charles Bronfman Institute for Personalized Medicine, Genetics of Obesity & Related Metabolic Traits Program, Icahn School of Medicine at Mount Sinai, One Gustave L. Levy Place, New York, NY 10029, USA. 11 Institute of Human Genetics, Helmholtz Zentrum München, German Research Center for Environmental Health, Ingolstädter Landstr. 1, 85764 Neuherberg, Germany. 12 Institut für Humangenetik, Technische Universität München, Trogerstr. 32, 81675 München, Germany. 13 Computational Medicine, Institute of Health Sciences, Faculty of Medicine, University of Oulu, Oulu, Finland. 14 Institute for Molecular Medicine Finland FIMM, University of Helsinki, Helsinki, Finland. 15 Department of Chronic Disease Prevention, National Institute for Health and Welfare, Helsinki, Finland. 16 Genetics of Complex Traits, University of Exeter Medical School, University of Exeter, Exeter, UK. 17 Wellcome Trust Centre for Human Genetics, Roosevelt Drive, Oxford OX3 7BN, UK. 18 Department of Oncology, Cancer and Haematology Centre, Churchill Hospital, Oxford, OX3 7LJ. 19 Singapore Immunology Network (SIgN), Agency for Science, Technology and Research (A*STAR), 8A Biomedical Grove, Singapore 138648. 20 University of Groningen, University Medical Center Groningen, Department of Gastroenterology and Hepatology, Hanzeplein 1, 9700RB, Groningen, The Netherlands. 21 Institute of Molecular and Cell Biology, University of Tartu, Riia 23, 51010 Tartu, Estonia. 22 Molecular Pathology, Institute of Biomedicine and Translational Medicine, University of Tartu, Ravila 19, Biomedicum, 50411, Tartu, Estonia. 23 Department of Epidemiology, Erasmus Medical Center Rotterdam, the Netherlands. 24 Institute for Clinical Chemistry and Laboratory Medcine, University Medicine Greifswald, Sauerbruchstr., 17475 Greifswald, Germany. 25 Institute for Community Medicine, University Medicine Greifswald, Walther-Rathenau-Str. 48, 17475 Greifswald, Germany. 26 Institute of Human Genetics, Helmholtz Zentrum München, German Research Center for Environmental Health, Ingolstädter Landstr. 1, 85764 Neuherberg, Germany. 27 Institut für Humangenetik, Technische Universität München, Trogerstr. 32, 81675 München, Germany. 28 Munich Heart Allience, Munich, Germany. 29 German Center for Cardiovascular Research (DZHK), Germany. 30 Institute for Clinical Diabetology, German Diabetes Center, Leibniz Center for Diabetes Research at Heinrich Heine University Düsseldorf, Auf'm Hennekamp 65, 40225 Düsseldorf, Germany. 31 German Center for Diabetes Research (DZD), partner site Düsseldorf, Germany. 32 Department of Diabetology and Endocrinology, University Hospital Düsseldorf, Heinrich Heine University, Moorenstr. 5, 40225 Düsseldorf, Germany. 33 Research Unit of Molecular Epidemiology, Helmholtz Zentrum München, German Research Center for Environmental Health, Ingolstädter Landstr. 1, 85764 Neuherberg, Germany. 34 Institute for Molecular Medicine Finland FIMM, University of Helsinki, Helsinki, Finland. 35 Department of Chronic Disease Prevention, National Institute for Health and Welfare, Helsinki, Finland. 36 Wellcome Trust Sanger Institute, Hinxton, Cambridge, United Kingdom. 37 Department of Public Health, Hjelt Institute, University of Helsinki, Helsinki, Finland. 38 Institute of Biomedical and Clinical Sciences, University of Exeter Medical School, Barrack Road, Exeter, EX2 5DW, UK. 39 Clinical Research Branch, National Institute on Aging NIA-ASTRA Unit, Harbor Hospital, MD, USA. 40 Laboratory of Neurogenetics, National Institute on Aging, National Institutes of Health, 35 Lincoln Drive, Bethesda, MD, USA. 41 Department of Molecular Neuroscience and Reta Lila Laboratories, Institute of Neurology, UCL, Queen Square House, Queen Square, London WC1N 3BG, UK. 42 Doctoral School in Translational and Molecular Medicine (DIMET), University of Milano-Bicocca, Piazza della Scienza, 3, 20126 Milan, Italy. 43 Department of Otolaryngology, National University of Singapore, Singapore. 44 Department of Neurology, Rudolf Magnus Institute of Neuroscience, University Medical Centre Utrecht, Utrecht, The Netherlands. 45 Institute of Genetic Epidemiology, Helmholtz Zentrum München, German Research Center for Environmental Health, Ingolstädter Landstr. 1, 85764 Neuherberg, Germany. 46 Institute of Medical Informatics, Biometry and Epidemiology, Chair of Genetic Epidemiology, Ludwig-Maximilians-Universität, Ingolstädter Landstr. 1, 85764 Neuherberg, Germany. 47 These authors contributed equally to this work. 48 These authors jointly directed this work. 49 Corresponding author (lude@ludesign.nl).

# Supplementary Note

## Setup of study

This eQTL meta-analysis is based on gene expression intensities measured in whole blood samples. RNA was isolated with either PAXgene Tubes (Becton Dickinson and Co., Franklin Lakes, NJ, USA) or Tempus Tubes (Life Technologies). To measure gene expression levels, Illumina Whole-Genome Expression Beadchips were used (HT12-v3 and HT12-v4 arrays, Illumina Inc., San Diego, USA). Although different identifiers are used across these different platforms, many probe sequences are identical. Meta-analysis could thus be performed if probe-sequences were equal across platforms. Integration of these probe sequences was performed as described before[1]. Genotypes were harmonized using HapMap2-based imputation using the Central European population[2]. In total, the eQTL genotype x environment interaction meta-analysis was performed on seven independent cohorts, comprising a total of 5,863 unrelated individuals. Mix-ups between gene expression samples and genotype samples were corrected using *MixupMapper*[3]. Gene expression normalization was performed as described before[4], removing up to 40 principal components (PCs). Additionally, we corrected for possible confounding factors due to arrays of poor RNA quality, by correlating the sample gene expression measurements against the first PC that was determined from the sample correlation matrix. Samples with a correlation < 0.9 were removed from further analysis.

## Participating cohorts

Informed consent was obtained from each participant, and the medical ethics committee of each of the participating cohorts. Statements of consent have been published together with each of the specific cohorts, and thus will not be repeated here.

### Fehrmann

The Fehrmann dataset consists of whole peripheral blood samples of 1,240 unrelated individuals from the UK and the Netherlands[5,6]. Some of these individuals are patients, while others are healthy controls. Individuals were genotyped using Illumina HumanHap300, HumanHap370 or the 610 Quad platform. Genotypes were imputed using Impute v2[7], using the phased genotypes of the CEU subpopulation of HapMap2 release 24 as reference[2]. RNA levels were quantified using the HT12v3 platform (N = 1,240), as has been described before[5]. The Fehrmann expression dataset is available at GEO (Gene Expression Omnibus) public repository under the accession ID GSE20142. As sample mix-up correction was performed prior to the participation in this study, the total number of samples with both genotype and gene-expression data, was equal to 1,240. After removing the gene expression samples which showed a poor RNA quality (correlation with PC1 < 0.9), 1,220 samples remained.

### SHIP-TREND

SHIP (Study of Health in Pomerania, Northeastern Germany) is a population-based project consisting of two independent cohorts, SHIP and SHIP-TREND. The study design of SHIP has been previously described in detail[8]. For this eQTL analysis, a part of the the SHIP-TREND cohort was used. The SHIP-TREND probands (N=986) were genotyped using the Illumina HumanOmni2.5-Quad arrays. Genotypes were imputed to HapMap v2[2] using IMPUTE[7]. RNA was prepared from whole blood collected under fasting conditions in PAXgene tubes (Becton Dickinson) using the PAXgene Blood miRNA Kit (Qiagen, Hilden, Germany). For SHIP-TREND this was done on a QIAcube according to protocols provided by the manufacturer (Qiagen). RNA was amplified (Ambion TotalPrep RNA), and hybridized to Illumina HumanHT-12 v3 Expression BeadChip. The SHIP-TREND expression dataset is available at GEO (Gene Expression Omnibus) public repository under the accession GSE 36382. After sample mix-up correction both imputed genotypes and whole-blood gene expression data were available for a total of 963 SHIP-TREND samples. No samples had to be removed due to poor RNA quality (correlation with PC1 < 0.9).

### Rotterdam Study

The Rotterdam Study (RS) is a large prospective, population-based cohort study in the district of Rotterdam, the Netherlands, investigating the prevalence, incidence, and risk factors of various chronic disabling diseases among elderly Caucasians aged 45 years and over. The initial cohort, named the Rotterdam Study I (or RS-I) was started in 1989, and consisted of 7,983 persons aged 55 years or over, living in the well-defined Ommoord district. In 1999, a second cohort, named the Rotterdam Study II (or RS-II) was started and consisted of 3,011 participants who had reached the age of 55 years since 1989 or who had moved into the study district. In 2006, the cohort was further extended with 3,932 subjects who were aged 45 years or over; this is called the Rotterdam Study III (RS-III). The Rotterdam Study has been described in detail[9,10]. Informed consent was obtained from each participant, and the medical ethics committee of the Erasmus Medical Center Rotterdam approved the study.

For this eQTL analysis, we used the RS-III cohort was used. The RS participants (n=3,054) were genotyped using the Illumina 610K quad arrays, and genotypes were imputed using MACH[11] with the HapMap CEU Phase 2 genotypes (release #22, build 36) as a reference[2]. Whole blood of 768 samples was collected (PAXgene Tubes, Becton Dickinson) and total RNA was isolated (PAXgene Blood RNA kit, Qiagen). RNA was amplified, labeled (Ambion TotalPrep RNA), and hybridized to the Illumina Whole-Genome Expression Beadchips (Human HT-12v4). The RS-III expression dataset is available at GEO (Gene Expression Omnibus) public repository under the accession GSE 33828. The total number of RS-III samples with both imputed genotypes and whole-genome expression data is equal to 768 (before sample mix-up correction). After sample mix-up correction, 762 samples remained. After removing the gene expression samples which showed a poor RNA quality (correlation with PC1 < 0.9), 755 samples remained.

### EGCUT

The Estonian Gene Expression Cohort[12] is composed of 899 samples (Mean age 37 [16.6] years; 50% females) from the Estonian Genome Center, University of Tartu (EGCUT) biobank cohort of 53,000 samples. Genotyping was performed using Illumina Human370CNV arrays (Illumina Inc., San Diego, USA), and imputed using Impute v2[7], using the HapMap CEU phase 2[2] genotypes (release #24, build 36). Whole peripheral blood RNA samples were collected using the Tempus Blood RNA Tubes (Life Technologies), and RNA was extracted using Tempus Spin RNA Isolation Kit (Life Technologies). Quality was measured by NanoDrop 1000 Spectrophotometer (Thermo Fisher Scientific, DE, USA) and Agilent 2100 Bioanalyzer (Agilent Technologies, CA, USA). Whole-Genome gene-expression levels were obtained by Illumina Human HT12v3 arrays (Illumina Inc, San Diego, CA, USA) according to the manufacturer’s protocols. After sample mix-up correction, 8 samples were excluded, and 891 samples remained. We did not have to remove any gene expression samples because of poor RNA quality (correlation with PC1 < 0.9), 891 samples remained.

### DILGOM

The Finnish study samples included a total of 513 unrelated individuals aged 25–74 years from the Helsinki area, recruited during 2007 as part of the Dietary, Lifestyle, and Genetic determinants of Obesity and Metabolic syndrome (DILGOM) study, an extension of the FINRISK 2007 study[13]. Study participants were asked to fast overnight (at least 10 hours) prior to giving a blood sample. DNA was extracted from 10 ml EDTA whole blood samples with salt precipitation method using Autopure (Qiagen GmbH, Hilden, Germany). DNA purity and quantity were assessed with PicoGreen (Invitrogen, Carlsbad, CA, USA) and genotyping used 250 ng of DNA which proceeded on the Illumina 610-Quad SNP array (Illumina Inc., San Diego, CA, USA) using standard protocols. SNPs were imputed with MACH version 1.0.10[11] using HapMap2 release 22[2] as a reference panel. To obtain stabilized total RNA, we used the PAXgene Blood RNA System (PreAnalytiX GMbH, Hombrechtikon, Switzerland). It included collection of 2.5 ml peripheral blood into PAXgene Blood RNA Tubes (Becton Dickinson and Co., Franklin Lakes, NJ, USA) and total RNA extraction with PAXgene Blood RNA Kit (Qiagen GmbH, Hilden, Germany). Protocol recommended by the manufacturer was used. The integrity and quantity of the RNA samples were evaluated with the 2100 Bioanalyzer (Agilent Technologies, Santa Clara, CA, USA). Biotinylated cRNA was produced from 200 ng of total RNA with Ambion Illumina TotalPrep RNA Amplification Kit (Applied Biosystems, Foster City, CA, USA), using the protocol specified by the manufacturer. 750 ng of biotinylated cRNA were hybridized onto Illumina HumanHT-12v3 Expression BeadChips (Illumina Inc., San Diego, CA, USA), using standard protocol (ArrayExpress database: accession number E-TABM-1036). After sample mix-up correction, 509 samples were included for further analysis in this cohort. After removing one gene expression sample which showed a poor RNA quality (correlation with PC1 < 0.9), 508 samples remained.

### InCHIANTI

InCHIANTI[14] is a population-based, prospective study in the Chianti area (Tuscany) of Italy. The participants were enrolled in 1998-2000, and were interviewed and examined every three years. Ethical approval was granted by the Instituto Nazionale Riposo e Cura Anziani institutional review board in Italy. Participants gave informed consent. Genome-wide genotyping was performed using the Illumina Infinium HumanHap550 genotyping chip. We used MACH 1.0.16[11] to impute using the HapMap r22 build-36 reference panel[2]. In the InCHIANTI study, peripheral blood specimens were taken using the PAXgene system (PreAnalytiX GMbH, Hombrechtikon, Switzerland), to preserve transcript expression levels. Samples were collected in 2008/9 (wave 4) from 712 participants and mRNA was extracted using the PAXgene Blood mRNA kit (Qiagen, Crawley, UK) according to the manufacturer’s instructions. Whole genome expression profiling of the samples was conducted using the Illumina Human HT-12 v3 microarray (Illumina, San Diego, CA, USA) as previously described[15]. Sample mix-up analysis on 620 samples passing QC and having both genotype and gene-expression data, revealed a total of 9 possible sample mix-ups. The total number of InCHIANTI samples with both imputed genotypes and whole-genome expression data included in this analysis was 611. After removing the gene expression samples which showed a poor RNA quality (correlation with PC1 < 0.9), 606 samples remained.

### KORA F4

KORA F4 (Cooperative Heath Research in the Region of Augsburg, Southern Germany) is a follow-up survey (2006-2008) of the population-based KORA S4 survey that was conducted in the region in 1999-2001. The expression analysis in this study was based on whole blood samples of the KORA F4 participants aged 62 to 81 year[16,17]. RNA was isolated from whole blood using PAXgene Blood miRNA Kit (Qiagen, Hilden, Germany). Purity and integrity of the RNA was analyzed using the Agilent Bioanalyzer with the 6000 Nano LabChip reagent set (Agilent Technologies, Germany). RNA was reverse transcribed with TotalPrep-96 RNA Amp Kit (Ambion, Germany) and hybridized to the Illumina HumanHT-12 v3 Expression BeadChip[17]. The samples were genotyped on the Affymetrix 6.0 GeneChip array[18]. The SNPs were imputed with MACH (v1.0.15)[11] and the HapMap CEU version 22[2] was used as reference population for calling and imputation. Altogether there were 740 samples with gene expression and genotype data available for analysis. We did not have to remove any gene expression samples because of poor RNA quality (correlation with PC1 < 0.9).

## Replication cohorts

### Stranger LCL

The Stranger Lymphoblastoid Cell Line (LCL) dataset consists of 608 individuals from HapMap3[19], which were hybridized to Illumina WG6v2 bead chips (ArrayExpress ID: E-MTAB-264). As genotypes, we used HapMap3 release 2 and gene expression measurements were normalized per population, using log_2_ transformation, quantile normalization, and principal component (PC) correction. PC correction was limited to 10 PCs because of the small sample size of each individual population. The LCL *cis*-eQTLs were subsequently meta-analyzed over all populations.

### CD4+ and CD8+ T-cells

Genotypes of this replication cohort are part of the EGCUT study (which is one of the discovery cohorts). As such, they have been generated using the same protocol and quality control measures.

#### Purification of CD4^+^ and CD8^+^ T cells

Peripheral blood was obtained from healthy donors of the Estonian Genome Center of the University of Tartu. Peripheral blood mononuclear cells (PBMC) were extracted using Ficoll-Paque (GE Healthcare) gradient centrifugation. CD4^+^ T cells and CD8^+^ T cells were extracted from the PBMCs by consecutive positive separation using microbeads (CD4+ #130-045-101; CD8+ #130-045-201) and AutoMACS technology (Miltenyi Biotec) according to the manufacturer's protocol. The study was approved by the Ethics Review Committee on Human Research of the University of Tartu, and all of the participants have signed a written informed consent.

#### RNA extraction, labelling and hybridization

RNA was extracted using the miRNeasy Mini Kit combined with a recommended RNase-free DNase I treatment (both from Qiagen) according to the manufacturer’s protocol. The RNA was labeled and amplified using the TargetAmp Nano Labeling Kit for Illumina Expression BeadChip (Epicentre Biotechnologies) with SuperScript III Reverse Transcriptase (Life Technologies) according to the manufacturer's protocol, followed by purification with the RNeasy MinElute Cleanup Kit (Qiagen). The labelled RNA samples were hybridized to HumanHT-12 v4 Expression BeadChips (Illumina) according to the manufacturer's instructions. The final number of individuals for this study was 309.

### Singapore Chinese functional genomics cohort

The Singapore Chinese cohort used for the neutrophil specific eQTL dataset is part of a larger epidemiological cohort described previously[20]. The individuals gave fresh whole blood which was used to isolate neutrophils and frozen down immediately in Trizol to -80°C. RNA was then extracted from this pure neutrophil population using the Ambion RNA exaction kit. The extracted RNA was then hybridized onto Illumina HumanHT-12 whole-genome gene expression chips. To avoid batch effects the RNA samples were randomly placed onto Illumina HumanHT-12 arrays such that each chip contains a number of samples from the various RNA extraction batches. Illumina microarray data was then normalized in Genome Studio using quantile normalization with no background subtraction. The log_2_ transformed expression values were then used for subsequent analysis. Additionally whole genome genotyping was done on these individuals using the Human Illumina Omni 5M chip. Samples were then checked for any pair of samples identified as first-degree relatives, and if found, these were removed. SNPs which were monomorphic in the population and those, which failed a call rate of 95% were also removed. This resulted in a total of 4.28 million SNPs with a good call rate that was taken forward for further statistical analysis. Thus, this batch of 114 samples was used to validate that the predicted eQTLs were indeed neutrophil specific.
 
Statistical analysis
Each significant SNP-probe pair in the discovery cohort was analyzed for significance in the validation cohort using a linear regression model. Array address IDs were matched to Illumina probe IDs using the annotation file HumanHT-12 v3. Linear regression was performed using the python function linregress from the library SciPy, coding the genotypes based on allele counts (0, 2 for homozygous genotypes and 1 for heterozygous genotypes). Samples with missing genotypes for a particular SNP were excluded from the analysis of the corresponding SNP-probe pair. 1000 genomes Illumina SNP IDs were mapped to rs IDs based on their annotated position on the chromosome.

### Oxford

Oxford cell-specific eQTL analysis has been described previously[21]. In the initial analysis peripheral blood mononuclear cell fractions were purified from 50ml of freshly collected EDTA anti-coagulated blood from 288 healthy European volunteers using Ficoll gradients. CD14+ monocytes and CD19+ B-cells were subsequently positively selected from this fraction using magnetic beads (MACS, Miltenyi-Bitotec, Bergisch Gladbach, Germany) with all steps performed on ice as per the manufacterer’s protocol. Individuals were genotyped at 730,525 markers using Illumina OmniExpress Beadchips and, after controlling for population outliers, 283 individuals were used in the final analysis. Genotypes were imputed using the CEU panel of HapMap2 release 24[2] using BEAGLE v2[22]. The final sample size for B-cells was 282 and the final sample size for monocytes was 283. The expression dataset is available at ArrayExpress public repository under the accession E-MTAB-945.

## Acknowledgements

### DILGOM

J.K. and S.R. were supported by funds from The European Community's Seventh Framework Programme (FP7/2007-2013) BioSHaRE, grant agreement 261433, S.R. was supported by funds from The European Community's Seventh Framework Programme (FP7/2007-2013) ENGAGE Consortium, grant agreement HEALTH-F4-2007- 201413", the Academy of Finland Center of Excellence in Complex Disease Genetics (grants 213506 and 129680), Academy of Finland (grant 251217), the Finnish foundation for Cardiovascular Research and the Sigrid Juselius Foundation. V.S. was supported by the Academy of Finland, grant number 139635 and Finnish Foundation for Cardiovascular Research. MP was partly financially supported for this work by the Finnish Academy SALVE program ‘‘Pubgensense’’ 129322 and by grants from the Finnish Foundation for Cardiovascular Research. The DILGOM-study was supported by the Academy of Finland, grant # 118065.

### SHIP-TREND

SHIP is part of the Community Medicine Research net of the University of Greifswald, Germany, which is funded by the Federal Ministry of Education and Research (grants no. 01ZZ9603, 01ZZ0103, and 01ZZ0403), the Deutsche Forschungsgemeinschaft (DFG GRK840-D2), the Ministry of Cultural Affairs as well as the Social Ministry of the Federal State of Mecklenburg-West Pomerania, and the network ‘Greifswald Approach to Individualized Medicine (GANI_MED)’ funded by the Federal Ministry of Education and Research (grant 03IS2061A). Genome-wide data have been supported by the Federal Ministry of Education and Research (grant no. 03ZIK012) and a joint grant from Siemens Healthcare, Erlangen, Germany and the Federal State of Mecklenburg, West Pomerania. Whole-body MR imaging was supported by a joint grant from Siemens Healthcare, Erlangen, Germany and the Federal State of Mecklenburg West Pomerania. The University of Greifswald is a member of the 'Center of Knowledge Interchange' program of the Siemens AG and the Caché Campus program of the InterSystems GmbH. The SHIP authors thank Mario Stanke for the opportunity to use his Server Cluster for the SNP imputation.

### EGCUT

EGCUT received financing by FP7 grants (201413, 245536), also received targeted financing from the Estonian Government (SF0180142s08) and direct funding from the Ministries of Research and Science and Social Affairs. EGCUT studies are funded by the University of Tartu in the framework of the Center of Translational Genomics and by the European Union through the European Regional Development Fund, in the framework of the Centre of Excellence in Genomics. We thank EGCUT personnel, especially Ms. M. Hass and Mr V. Soo. EGCUT data analyses were carried out in part in the High Performance Computing Center of the University of Tartu.

### Rotterdam Study

We thank Pascal Arp, Mila Jhamai, Marijn Verkerk, Lizbeth Herrera, and Marjolein Peters for their help in creating the GWAS database; Karol Estrada and Maksim Struchalin for their support in creation and analysis of imputed data; Tobias A. Knoch, Anis Abuseiris, Karol Estrada, and Rob de Graaf as well as their institutions, the Erasmus GRID Office, Erasmus MC Rotterdam, The Netherlands, and especially the national German MediGRID and Services@MediGRID part of the German D-Grid, both funded by the German Bundesministerium fuer Forschung und Technology under grants #01 AK 803 A-H and # 01 IG 07015 G for access to their grid resources. The authors thank the study participants and staff from the Rotterdam Study, the participating general practitioners and the pharmacists.

The Rotterdam Study was funded by the European Commission (HEALTH-F2-2008-201865, GEFOS; HEALTH-F2-2008 35627, TREAT-OA 200800), the Netherlands Organization of Scientific Research NWO Investments (nos 175.010.2005.011, 911-03-012), the Research Institute for Diseases in the Elderly (014-93-015; RIDE2), the Netherlands Genomics Initiative (NGI)/Netherlands Consortium for Healthy Aging (NCHA) (project nr. 050-060-810), an NWO VIDI grant (#917103521).

The Rotterdam Study is funded by Erasmus Medical Center and Erasmus University, Rotterdam, Netherlands Organization for Health Research and Development (ZonMw), the Research Institute for Diseases in the Elderly (RIDE), the Ministry of Education, Culture and Science, the Ministry for Health, Welfare and Sports, the European Commission (DG XII), and the Municipality of Rotterdam.

### Fehrmann

L.F.,H-J.W.: This study was supported by grants from the Celiac Disease Consortium (an innovative cluster approved by the Netherlands Genomics Initiative and partly funded by the Dutch Government (grant BSIK03009), the Netherlands Organization for Scientific Research (NWO-VICI grant 918.66.620, NWO-VENI grant 916.10.135 to L.F.), the Dutch Digestive Disease Foundation (MLDS WO11-30), and a Horizon Breakthrough grant from the Netherlands Genomics Initiative (grant 92519031 to L.F.). This project was supported by the Prinses Beatrix Fonds, VSB fonds, H. Kersten and M. Kersten (Kersten Foundation), The Netherlands ALS Foundation, and J.R. van Dijk and the Adessium Foundation. The research leading to these results has received funding from the European Community’s Health Seventh Framework Programme (FP7/2007-2013) under grant agreement 259867. We especially thank Jackie Senior and Kate McIntyre for critically reading the manuscript. This study was supported by the BBMRI NL Functional Genomics Project. Funding for the project was provided by the Netherlands Organization for Scientific Research under award number 184021007, dated July 9, 2009 and made available as a Rainbow Project of the Biobanking and Biomolecular Research Infrastructure Netherlands (BBMRI–NL). D.A. was supported by the Centre for BioSystems Genomics (CBSG) and the Netherlands Consortium of Systems Biology (NCSB), both of which are part of the Netherlands Genomics Initiative / Netherlands Organisation for Scientific Research.

### InCHIANTI

InCHIANTI was supported by the Wellcome Trust 083270/Z/07/Z. The InCHIANTI study was supported by contract funding from the U.S. National Institute on Aging (NIA), and the research was supported in part by the Intramural Research Program, NIA, and National Institute of Health (NIH). A.R.W. was supported by the Peninsula NIHR Clinical Research Facility. Funding to pay the Open Access publication charges for this article was provided by the Wellcome Trust.

### KORA F4

The KORA authors acknowledge the contributions of Peter Lichtner, Gertrud Eckstein, Guido Fischer, Norman Klopp, Nicole Spada, and all members of the Helmholtz Zentrum München genotyping staff for generating the SNP data and Katja Junghans and Anne Löschner (Helmholtz Zentrum München) for generating gene expression data from both KORA and SHIP-TREND samples.
The KORA research platform and the KORA Augsburg studies are financed by the Helmholtz Zentrum München, German Research Center for Environmental Health, which is funded by the BMBF and by the State of Bavaria. We thank the field staff in Augsburg who were involved in the studies. The German Diabetes Center is funded by the German Federal Ministry of Health and the Ministry of School, Science and Research of the State of North-Rhine-Westphalia. The Diabetes Cohort Study was funded by a German Research Foundation project grant to W.R. (DFG; RA 459/2-1). This study was supported in part by a grant from the BMBF to the German Center for Diabetes Research (DZD e.V.), by the DZHK (Deutsches Zentrum für Herz-Kreislauf-Forschung – German Centre for Cardiovascular Research) and by the BMBF funded Systems Biology of Metabotypes grant (SysMBo#0315494A). Additional support was given by the BMBF (National Genome Research Network NGFNplus Atherogenomics, 01GS0834) and the Leibniz Association (WGL Pakt für Forschung und Innovation). We thank Maren Carstensen, Gabi Gornitzka and Astrid Hoffmann (German Diabetes Center) for excellent technical assistance.

### Oxford

This work was supported by the Wellcome Trust (Grants 074318 [J.C.K.], 088891 [B.P.F.], and 075491/Z/04 [core facilities Wellcome Trust Centre for Human Genetics]), the European Research Council under the European Union’s Seventh Framework Programme (FP7/2007-2013) / ERC Grant agreement no. 281824 (J.C.K.) and the NIHR Oxford Biomedical Research Centre.

### Singapore Chinese functional genomics cohort

These studies were supported by A*STAR/SIgN core funding, and grants SIgN-06-006, SIgN-08-020 and SIgN-10-029.

## References

1. Westra H-J, Peters MJ, Esko T, Yaghootkar H, Schurmann C, et al. (2013) Systematic identification of trans eQTLs as putative drivers of known disease associations. Nat Genet 45: 1238–1243. doi:10.1038/ng.2756.

2. The International HapMap Project (2003). Nature 426: 789–796. doi:10.1038/nature02168.

3. Westra H-J, Jansen RC, Fehrmann RSN, Te Meerman GJ, Van Heel D, et al. (2011) MixupMapper: correcting sample mix-ups in genome-wide datasets increases power to detect small genetic effects. Bioinformatics 27: 2104–2111. doi:10.1093/bioinformatics/btr323.

4. Westra H-J, Peters MJ, Esko T, Yaghootkar H, Schurmann C, et al. (2013) Systematic identification of trans eQTLs as putative drivers of known disease associations. Nat Genet. doi:10.1038/ng.2756.

5. Fehrmann RSN, Jansen RC, Veldink JH, Westra H-J, Arends D, et al. (2011) Trans-eQTLs reveal that independent genetic variants associated with a complex phenotype converge on intermediate genes, with a major role for the HLA. PLoS Genet 7: e1002197. doi:10.1371/journal.pgen.1002197.

6. Dubois PCA, Trynka G, Franke L, Hunt KA, Romanos J, et al. (2010) Multiple common variants for celiac disease influencing immune gene expression. Nat Genet 42: 295–302. doi:10.1038/ng.543.

7. Howie BN, Donnelly P, Marchini J (2009) A flexible and accurate genotype imputation method for the next generation of genome-wide association studies. PLoS Genet 5: e1000529. doi:10.1371/journal.pgen.1000529.

8. Völzke H, Alte D, Schmidt CO, Radke D, Lorbeer R, et al. (2011) Cohort profile: the study of health in Pomerania. Int J Epidemiol 40: 294–307. doi:10.1093/ije/dyp394.

9. Hofman A, van Duijn CM, Franco OH, Ikram MA, Janssen HLA, et al. (2011) The Rotterdam Study: 2012 objectives and design update. Eur J Epidemiol 26: 657–686. doi:10.1007/s10654-011-9610-5.

10. Hofman A, Darwish Murad S, van Duijn CM, Franco OH, Goedegebure A, et al. (2013) The Rotterdam Study: 2014 objectives and design update. Eur J Epidemiol 28: 889–926. doi:10.1007/s10654-013-9866-z.

11. Li Y, Willer CJ, Ding J, Scheet P, Abecasis GR (2010) MaCH: using sequence and genotype data to estimate haplotypes and unobserved genotypes. Genet Epidemiol 34: 816–834. doi:10.1002/gepi.20533.

12. Metspalu A (2004) The Estonian Genome Project. Drug Dev Res 62: 97–101. doi:10.1002/ddr.10371.

13. Inouye M, Silander K, Hamalainen E, Salomaa V, Harald K, et al. (2010) An immune response network associated with blood lipid levels. PLoS Genet 6: e1001113. doi:10.1371/journal.pgen.1001113.

14. Tanaka T, Shen J, Abecasis GR, Kisialiou A, Ordovas JM, et al. (2009) Genome-wide association study of plasma polyunsaturated fatty acids in the InCHIANTI Study. PLoS Genet 5: e1000338. doi:10.1371/journal.pgen.1000338.

15. Gibbs JR, van der Brug MP, Hernandez DG, Traynor BJ, Nalls MA, et al. (2010) Abundant quantitative trait loci exist for DNA methylation and gene expression in human brain. PLoS Genet 6: e1000952. doi:10.1371/journal.pgen.1000952.

16. Rathmann W, Strassburger K, Heier M, Holle R, Thorand B, et al. (2009) Incidence of Type 2 diabetes in the elderly German population and the effect of clinical and lifestyle risk factors: KORA S4/F4 cohort study. Diabet Med 26: 1212–1219. doi:10.1111/j.1464-5491.2009.02863.x.

17. Mehta D, Heim K, Herder C, Carstensen M, Eckstein G, et al. (2013) Impact of common regulatory single-nucleotide variants on gene expression profiles in whole blood. Eur J Hum Genet 21: 48–54. doi:10.1038/ejhg.2012.106.

18. Marzi C, Albrecht E, Hysi PG, Lagou V, Waldenberger M, et al. (2010) Genome-wide association study identifies two novel regions at 11p15.5-p13 and 1p31 with major impact on acute-phase serum amyloid A. PLoS Genet 6: e1001213. doi:10.1371/journal.pgen.1001213.

19. Stranger BE, Montgomery SB, Dimas AS, Parts L, Stegle O, et al. (2012) Patterns of cis regulatory variation in diverse human populations. PLoS Genet 8: e1002639. doi:10.1371/journal.pgen.1002639.

20. Andiappan AK, Wang DY, Anantharaman R, Parate PN, Suri BK, et al. (2011) Genome-wide association study for atopy and allergic rhinitis in a Singapore Chinese population. PLoS One 6: e19719. doi:10.1371/journal.pone.0019719.

21. Fairfax BP, Makino S, Radhakrishnan J, Plant K, Leslie S, et al. (2012) Genetics of gene expression in primary immune cells identifies cell type-specific master regulators and roles of HLA alleles. Nat Genet 44: 502–510. doi:10.1038/ng.2205.

22. Browning BL, Browning SR (2009) A unified approach to genotype imputation and haplotype-phase inference for large data sets of trios and unrelated individuals. Am J Hum Genet 84: 210–223. doi:10.1016/j.ajhg.2009.01.005.

23. Preininger M, Arafat D, Kim J, Nath AP, Idaghdour Y, et al. (2013) Blood-informative transcripts define nine common axes of peripheral blood gene expression. PLoS Genet 9: e1003362.

**Supporting Information Legends**

**S1 Fig Neutrophil percentage and gene expression correlation distribution in EGCUT**

In order to predict the neutrophil percentage, we selected 58 gene expression probes (0.1% of the dataset) that strongly positively correlated with neutrophil percentage in the EGCUT dataset (Spearman R > 0.57, P < 3 x 10^-72^, n = 825).

**S2 Fig Comparison of expression in the BLUEPRINT study**

The 58 probes we used to estimate neutrophil percentage map to 44 unique genes. We compared the gene expression levels for these genes using RNA-seq data from the BLUEPRINT consortium among 14 different cell types. For most of these cell-types multiple biological replicates have been assayed. We quantile normalized, log_2_ transformed and then centered the expression levels for every individual gene to a mean of zero and a standard deviation of one. We observed that 42 of these 44 genes show significantly higher expression (Student's T-test P < 0.001), as compared to the other 13 cell types. NS: non significant.

**S3 Fig Relationship between neutrophil percentage, age and gender**

We correlated the actual neutrophil percentage (top) and the inferred neutrophil percentage (bottom) with age in the EGCUT dataset (n = 825) and observed that there is a low, but significant correlation between age and neutrophil percentage. However, neutrophil percentage is not significantly associated with gender.

### S4 Fig Stability of neutrophil percentage prediction

We tested the stability of our neutrophil percentage prediction in the EGCUT dataset (n=825). From the list of 100 probes showing highest correlation with neutrophil percentage, we randomly selected a number of probes (increments of 5 probes, 1000 permutations per increment) and repeated the neutrophil percentage prediction. When including > 10 probes, the neutrophil prediction displays stable correlation with the actual neutrophil percentage (Spearman R ~0.75) and near perfect correlation with the predicted neutrophil percentage used in the meta-analysis (Spearman R ~0.99). Error bars denote standard deviation. Red line denotes the number of gene expression probes the different cohorts in this study used to estimate neutrophil percentage.

### S5 Fig *Cis*-eQTL effect size and cell type specificity

71% of the *cis*-eQTLs that were identified as being cell type specific by our method show an effect size larger than 0.03 in our original *cis*-eQTL meta-analysis (Westra *et al*, 2013), compared to 21% for those that do not have a significant interaction effect.

### S6 Fig Comparison of effect sizes and effect direction between datasets

Comparison of interaction effect Z-scores shows a high consistent direction of effect between datasets and with the meta-analysis for those interaction effects significant at FDR < 0.05.

**S7 Fig Relationship between *NOD2* gene expression levels, age and gender**

We correlated the actual *NOD2* gene expression levels with age in the EGCUT dataset (n = 825, normalized using log_2_ transformed and quantile normalization, and gene expression levels corrected for 40 principal components) and observed that there is a low, but significant correlation between age and *NOD2* gene expression in the log_2_ transformed and quantile normalized data (top), which becomes insignificant when correcting the gene expression data for 40 principal components (which was used to determine the neutrophil interaction effect; bottom). However, *NOD2* gene expression levels are not significantly associated with gender.

### S8 Fig Effect of robust estimation of standard errors

The interaction model we used does not take heteroscedasticity into account. Therefore, we determined standard errors using the 'sandwich' package in R, which allows for the estimation of robust standard errors. We observed strong correlation between standard errors, Z-scores and p-values by our model and a model that applies robust estimation of standard errors in the EGCUT (top) and Fehrmann datasets (bottom).

### S9 Fig Principal components on gene expression data

Principal component 1 (PC1) and principal component 2 per study. Samples with a correlation < 0.9 with PC1 (red) were excluded from analysis.

### S10 Fig Neutrophil percentage and principal component correction

The gene expression data that was used for the interaction meta-analysis was corrected for up to 40 principal components. In order to retain genetic variation in the gene expression data, components that showed a significant correlation with genotypes were not removed. In the EGCUT dataset (n = 825), many of these components also strongly correlate with neutrophil percentage (top) and inferred neutrophil percentage (bottom). The majority of the variation in gene expression explained by these components (right) was however removed from this dataset.

### S1 Table

List of 58 Illumina HT12v3 probes used for calculating the estimated neutrophil percentage principal component score and their correlation with neutrophil percentage in the EGCUT dataset (n = 825).

### S2 Table

Summary statistics for the interaction analysis.

### S3 Table

Results of the interaction analysis.

### S4 Table

Summary statistics showing the effect size (correlation coefficient) in each of the tested replication datasets.

### S5 Table

Results of the neutrophil mediated *cis*-eQTL disease enrichment analysis.

**S6 Table**

We created proxy phenotypes for the 9 axes of variation described by Preininger et al [23] within the EGCUT (n = 891) and Fehrmann (n = 1,220) cohorts. We then meta-analyzed the interaction terms for these two cohorts and observed that several axes mediate eQTL effects. The Z-scores for the interaction effects for axis 5 correlate strongly with the Z-scores for interaction effects for the neutrophil proxy (R = 0.74).
